# Revealing the reaction path of UVC bond rupture in cyclic disulfides with ultrafast x-ray scattering

**DOI:** 10.1126/sciadv.adp9175

**Published:** 2025-01-15

**Authors:** Lingyu Ma, Wenpeng Du, Haiwang Yong, Brian Stankus, Jennifer M. Ruddock, Andrés Moreno Carrascosa, Nathan Goff, Yu Chang, Nikola Zotev, Darren Bellshaw, Thomas J. Lane, Mengning Liang, Sébastien Boutet, Sergio Carbajo, Joseph S. Robinson, Jason E. Koglin, Michael P. Minitti, Adam Kirrander, Theis I. Sølling, Peter M. Weber

**Affiliations:** ^1^ Department of Chemistry, Brown University, Providence, RI, USA.; ^2^ Department of Chemistry and Biochemistry, University of California, San Diego, CA, USA.; ^3^ Department of Chemistry, Western Connecticut State University, Danbury, CT, USA.; ^4^ Physical and Theoretical Chemistry Laboratory, Department of Chemistry, University of Oxford, Oxford, UK.; ^5^ Linac Coherent Light Source, SLAC National Accelerator Laboratory, Menlo Park, CA, USA.; ^6^ Center for Integrative Petroleum Research, College of Petroleum & Geosciences, King Fahd University of Petroleum & Minerals, Dhahran 31261, Saudi Arabia.

## Abstract

Disulfide bonds are ubiquitous molecular motifs that influence the tertiary structure and biological functions of many proteins. Yet, it is well known that the disulfide bond is photolabile when exposed to ultraviolet C (UVC) radiation. The deep-UV–induced S─S bond fragmentation kinetics on very fast timescales are especially pivotal to fully understand the photostability and photodamage repair mechanisms in proteins. In 1,2-dithiane, the smallest saturated cyclic molecule that mimics biologically active species with S─S bonds, we investigate the photochemistry upon 200-nm excitation by femtosecond time-resolved x-ray scattering in the gas phase using an x-ray free electron laser. In the femtosecond time domain, we find a very fast reaction that generates molecular fragments with one and two sulfur atoms. On picosecond and nanosecond timescales, a complex network of reactions unfolds that, ultimately, completes the sulfur dissociation from the parent molecule.

## Teaser

Ultrafast x-ray scattering is used to probe the chemical reaction dynamics of a cyclic disulfide upon UVC exposure.

## INTRODUCTION

The S─S disulfide bond, a ubiquitous building block of molecular biology, plays a central role in creating and maintaining the secondary and tertiary structures of proteins (*1*). By forming covalent interactions between ligands and proteins, disulfide bonds determine the mechanisms of many cellular and immunological responses, including the stabilization of protein folding, the function of enzyme catalysis, and the protection against oxidative damages (*2*). Yet, typical disulfide bonds have binding energies that are smaller than the energies of visible or UV photons, raising important questions about the fate of biochemical structures after photoexcitation. In nucleotides, light-induced polymerization has been found to be an important avenue leading to cancer (*3*), although there are effective de-excitation routes (*4*) that rapidly convert the deleterious photon energy to thermal energy (*5*, *6*). The disulfide link has been shown to be photolabile (*7*). Yet, it has survived billions of years of evolution, including the harsh, pre-ozone layer eras of early life. This suggests that disulfide bond structures must have found ways to survive the high-energy UV radiation conditions or used the instability as an evolutionary advantage (*8*).

The behavior of the straight chain disulfide bond stands in contrast to that of cyclic disulfides, where a repulsive excited (S_1_) state drives the sulfur atoms apart and initiates a torsional motion of the carbon skeleton (*9*). The molecule 1,2-dithiane (DT) is a prototypical unstrained, saturated cyclic molecule with an S─S bond that mimics this important structural motif. A recent study showed that “soft” excitation of DT using 284-nm photons induces dynamics on picosecond timescales leading back to the original cyclic structure (*9*). This effect was supported by simulations that postulated a “Newton’s Cradle” motion with low-amplitude movements leading to the reformation of the disulfide bond (*10*). Higher-energy excitation by 200-nm photons leads to highly excited states, which compromises the structural integrity of the disulfide moiety, so that the original cyclic structure is lost on a longer timescale (*11*). This raises important questions about the fate of the molecule after deep-UV excitation. Specifically, will the formed biradical structures allow the system to recover its original disulfide bond, or is the bond irreversibly destroyed by the high photon energy?

Historically, spectroscopic experiments have generated a wealth of knowledge about photochemical reaction mechanisms (*9*, *11*), although spectra are indirect probes of structural dynamics, and their analysis is affected by poorly characterized parameters such as ionization cross sections and fragmentation probabilities. Recently, ultrafast x-ray free electron lasers (XFELs) and mega-electron-volt ultrafast electron diffraction (MeV-UED) have advanced the determination of transient molecular structures into a new era where interatomic distances can be measured with sub-Ångström spatial resolution and femtosecond time resolution (*12*–*17*). X-ray scattering signals directly interrogate the relative positions of nuclei, yielding detailed information about the transient structures during photochemical reactions. Furthermore, the high brightness XFELs have opened the door to study photochemistry in low-density gas phase vapors, where the reaction dynamics can unfold without the interference of nearby molecules. Moreover, the x-ray scattering intensities are quantitatively related to the number of electrons in the target (*18*), so that kinetic analyses can be performed without the deleterious effects of unknown scaling factors.

The focus of the present time-resolved x-ray scattering study is to explore the fate of DT upon excitation by deep-UV radiation. Previously, Ochmann *et al*. (*19*) have studied the photochemical kinetics of a linear disulfide, dimethyl disulfide, in solution upon excitation with 267-nm UV pulses using x-ray absorption spectroscopy at the sulfur *K*-edge. Fragmentation products beyond the sulfur radicals were found, revealing complex kinetics even for this smallest organic disulfide molecule at a comparatively low excitation energy. In the gas phase, the fragmentation of a linear disulfide chain structure makes a reformation of the disulfide bond nearly impossible. One expects that cyclic disulfides would exhibit fragmentation channels similar to those of the chain disulfides plus additional channels that arise from the confinement of the two sulfur radicals within close proximity during the intermediate stages of the reaction. The initial step of the dynamics of cyclic disulfide bond is well understood (*9*–*11*), but the kinetics beyond the non-ergodic stage is not. The goal of the present study is to explore the reaction kinetics and the full decomposition scheme of the cyclic dithiane after the initial dynamics through the electronic states.

**Fig. 1.**
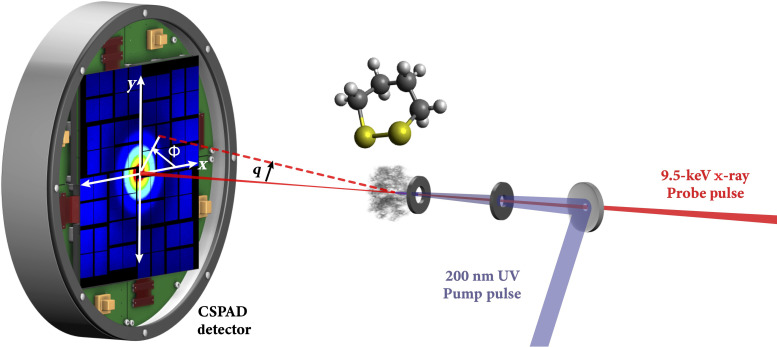
A schematic of the experiment. The photoinduced reaction of DT is initiated by a 200-nm UV pump pulse, and the time-evolving molecular structures of reactants and photoproducts are probed by scattering using 9.5-keV x-ray pulses with a variable time delay. The scattering signals are recorded with a CSPAD detector. Created in Adobe Illustrator.

The DT molecules are excited by 200-nm pump pulses into a manifold of high-lying electronic states. Scattering patterns are measured by intersecting the sample with 9.5-keV x-ray probe pulses from the Linac Coherent Light Source (LCLS) x-ray free electron lasers at SLAC National Laboratory while scanning the pump-probe time delay. The scattering signals were recorded by a 2.3-megapixel Cornell-SLAC pixel array detector (*20*) (CSPAD) shown in Fig. 1. The analysis of the time-dependent scattering signals reveals a complex reaction network that unfolds on time scales encompassing the full observation window, from the sub-picosecond to the nanosecond regime.

## RESULTS

The time-evolving scattering signals are expressed as percent differences

$$\%∆I\left(q,t\right)=100\cdot \frac{I_{\text{on}}\left(q,t\right)-I_{\text{off}}\left(q\right)}{I_{\text{off}}\left(q\right)}$$ (1)

where $I_{\text{on}}\left(q,t\right)$ is the scattering signal with the pump laser at a given momentum transfer *q* and delay time *t*, while $I_{\text{off}}\left(q\right)$ represents the scattering pattern of the ground-state, unexcited molecules. The linear polarization of the optical pump pulse induces an alignment in the molecular sample that gives rise to an anisotropy component of the scattering signal, related to the orientation of the molecular transition dipole moment with respect to the molecular axes (*21*). Both the isotropic and anisotropic components are retrieved from the total scattering signals using standard methods (*22*, *23*), but the present analysis focuses on the former, which more clearly reveals molecular structures involved in the photoinduced reaction kinetics of DT upon deep-UV excitation. On the basis of published absorption spectra, time-resolved photoelectron spectra, and theoretical simulations, S_5_ is the most likely initial state upon 200-nm excitation (*11*).

**Fig. 2.**
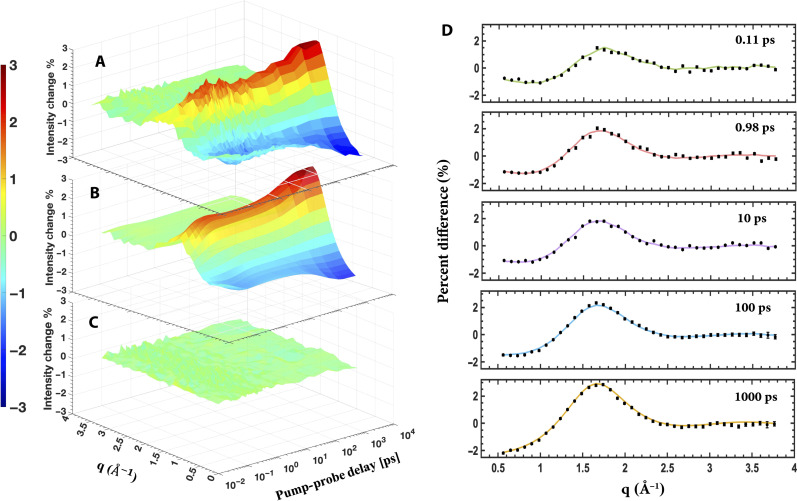
Comparison of experimental and simulated time-dependent percent difference scattering signals of the photoinduced chemical reaction of DT. (**A**) The isotropic component of the time-dependent experimental percent difference scattering signal of DT as a function of pump-probe time delays. Plotted is the percent difference in scattering intensity (color bar) induced by the laser pulse as a function of the absolute value of the momentum transfer vector, *q*, and the pump-probe time delay. (**B**) Simulated time-dependent percent difference scattering signal of DT based on the proposed kinetic model and kinetic analysis fitting results. (**C**) Residuals of the experimental percent difference scattering signals with respect to the simulated percent difference scattering signals as a function of the absolute value of the momentum transfer vector *q* and the pump-probe time delay. (**D**) Experimental pump-probe x-ray scattering percent difference signals (black dots with 3σ experimental noise) and kinetic fit results (colored lines) at selected representative time delays.

The isotropic component of the percent difference scattering signal (Fig. 2A) shows a time dependence that stretches from the femtosecond time regime to the 3-ns maximum time of the experiment. In the femtosecond domain, the excitation to the S_5_ state launches the structural dynamics (*11*). The femtosecond time domain scattering signals show the initially excited state and the photoproducts immediately following the electronic decay out of those high-lying excited states. The negative signal at low *q* in the sub-picosecond time regime immediately suggests the presence of ultrafast dissociation processes (*18*). Scattering patterns in the picosecond time domain, i.e., the patterns between 1 and 15 ps in Fig. 2A, reveal initial photochemical reaction transients. At longer time delays, from 15 to 3 ns, there are further dramatic changes in the percent difference scattering signals, suggesting consecutive kinetic reactions involving the intermediates.

As discussed above, the experiment records the scattering intensity as a function of two variables, the pump-probe delay time *t* and the momentum transfer *q*. Further analysis deconvolutes this two-dimensional signal into a probability of optical excitation (a scalar quantity), *q*-dependent scattering patterns of the reaction transients (which are determined from the multidimensional nonlinear fitting of the data), and the time-dependent kinetics of the chemical reaction sequence. The data provide a very tight constraint, allowing even the complicated reaction network of DT to be captured in full. As the reaction unfolds over time, the time-dependent percentages of the various transients must add up quantitatively. The magnitude of the x-ray scattering intensities of the transients is fully determined by the electron count and distribution of electrons in each molecular species. Because of this quantitative property of the x-ray scattering, the two-dimensional scattering signal is very deterministic (*24*). The excellent agreement between the experimental and fitted scattering signals shown in Fig. 2D confirms the validity of the kinetic scheme and yields scattering patterns for the individual reaction transients, which can then be used to identify these transients via comparison to simulations based on computed molecular structures.

**Fig. 3.**
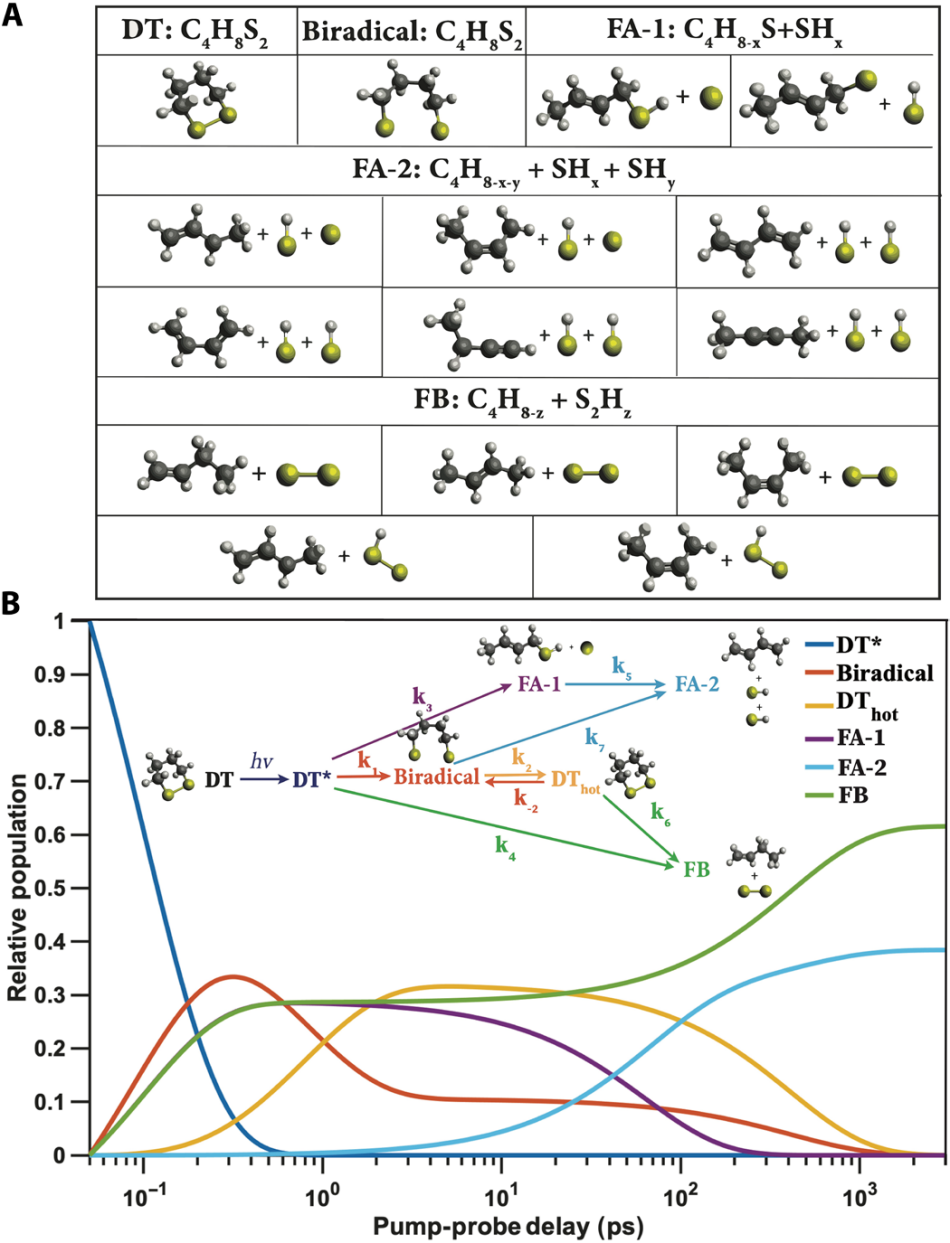
The kinetic model of the photoinduced chemical reaction of DT upon 200-nm excitation. (**A**) Illustrations of possible intermediate transients and photoproducts within the photoinduced chemical reaction network. The positions of hydrogen atoms remain undetermined because of the small x-ray scattering cross section for hydrogen. Several structures are possible for intermediate transient FA-1 and the final photoproducts FA-2 and FB as shown (see possible geometries in the “The structures of the photochemical reaction transients and photoproducts” section in Supplementary Text). (**B**) Time-dependent relative populations of transients and photoproducts. Inset: The photoinduced chemical reaction scheme.

To analyze the observed scattering patterns, we postulate a very general reaction scheme and let the experimental data define which pathways take place, what the time scales are, and the nature of the transients. The general reaction scheme of Fig. 3B (inset) is a highly flexible model that includes the possibility of ultrafast (femtosecond) isomerization and dissociative reactions and slower (picosecond to nanosecond) kinetic processes that lead to additional dissociations. More complicated schemes were tested but did not improve the quality of the fit. Simpler schemes that omit the important reaction paths of Fig. 3B did not yield adequate results.

In the proposed scheme, the pump photon excites the molecules to a manifold of electronically excited states, indicated as DT*. From there, one channel leads to the rupture of the disulfide bond and the creation of the biradical transient. Other reaction channels, denoted as A and B, with rate constants k_3_ and k_4_, lead to FA-1 (fragment A – intermediate) and FB (fragment B). These channels may be related to the dissociation along the repulsive S_2_ potential energy surface, and the S_5_ and S_5v_ channels observed in the photoelectron spectra (*11*). FA-1 is an intermediate on the way to FA-2 (fragment A – photoproduct), which is one of the final fragments of the photochemical reaction. Once the molecules arrive on the ground-state potential energy surface of the biradical and the energy is redistributed among vibrational modes, a fast equilibrium is established between the biradical and vibrationally hot dithiane (DT_hot_). From there, the fragmentation may lead from DT_hot_ to FB or from the biradical to FA-2. The FA-1 transients that are created early in the reaction can also react to FA-2. Although the small x-ray scattering cross section for hydrogen atoms made the determination of H atoms in the final fragments ambiguous, the analysis does determine the heavy-atom skeletons of the transients and final photoproducts. All possible placements of hydrogen atoms give satisfactory agreements with the experimental data (see section S2).

The percent difference scattering signals as a function of the pump-probe delay time *t* and the absolute value of the momentum transfer vector *q* (Fig. 2A) arises from the isotropic scattering patterns of the transient species (*q* dependence) and their corresponding time-dependent relative populations as determined by the kinetics scheme (the time dependence). Treating the scattering patterns for each species as adjustable functions, the measured isotropic percent difference scattering pattern is fitted by the equation (*25*)

$$\Delta I_{\text{iso}}\left(q,t\right)=\gamma \left[\sum_{\alpha }S_{\alpha }\left(q\right)F_{\alpha }\left(t\right)\right]$$ (2)

where the fraction of molecules that are optically excited (#x03B3;) is a scalar quantity that is determined in the overall experimental analysis. $F_{\alpha }\left(t\right)$ is the time-dependent relative population of species α obtained by solving the rate differential equations of the kinetic scheme. The *q*-dependent isotropic scattering patterns for species, α i.e., $S_{\alpha }\left(q\right)$, are obtained from the experimental data through the kinetics fits. The measured experimental signal is related to the time dependence of nuclear geometries and their corresponding x-ray scattering patterns. The tight constraint arising from both the time and momentum transfer dimension makes the measured signals highly deterministic for the kinetic scheme and its parameters. Comparisons with theoretical patterns of the possible transients (Fig. 3A) identify the particles involved in the reaction. More details about the kinetics fits are provided in the “Additional details about the kinetics fit” section in Supplementary Text.

The model provides an excellent fit with the resulting kinetic parameters shown in Table 1. Accordingly, the fraction of optically excited molecules is determined to be γ = 4.5%. During the experiment, the optical pump laser intensity was intentionally kept low to minimize the probability of multi-photon excitation processes that could induce alternate reaction processes (*26*). The time-dependent percent difference scattering signal of the DT dissociation reaction is shown in Fig. 2B. Comparison with the experimental data (Fig. 2A) results in small residuals (Fig. 2C), suggesting a good fit in both the time and momentum transfer dimensions. The residuals fluctuate randomly around the baseline as is expected for experimental noise, indicating that the data contain no additional information beyond the reaction scheme.

**Table 1.** The reaction rates and corresponding time constants for the proposed photochemical reaction mechanism.

Kinetic parameter	Reaction Rates, *k*_*x*_ (×10^12^s^−1^)	Time constants, 1/*k*_*x*_
*k*_1_	4.23 ± 0.09	240 ± 10 fs
*k*_2_	0.95 ± 0.04	1.05 ± 0.05 ps
*k*_−2_	0.31 ± 0.04	3.20 ± 0.44 ps
*k*_3_	2.86 ± 0.06	350 ± 10 fs
*k*_4_	2.85 ± 0.06	350 ± 10 fs
*k*_5_	0.0158 ± 2.5 × 10^−5^	63 ± 0.10 ps
*k*_6_	0.0025 ± 0.1 × 10^−5^	400 ± 0.16 ps
*k*_7_	0.0022 ± 0.4 × 10^−5^	455 ± 0.74 ps

Figure 3B shows the time-dependent relative population $F_{\alpha }\left(t\right)$ of each species during the photochemical reaction on a logarithmic timescale. In the femtosecond time domain, the decay of DT* and the rise of the biradical, FA-1, and FB dominate. Although this initial decay process is likely of dynamic nature, the data did not uncover concerted wave packet motions. Consequently, the decays were modeled using kinetic parameters (see Table 1). The very similar reaction rates $k_{1}$, $k_{3}$, and $k_{4}$ are well determined by the relative product populations of the parallel reaction channels. The early picosecond time domain is a metastable stage that involves the thermalization of the energy and the establishment of a fast equilibrium between the biradical and DT_hot_. At later times, from several hundred picoseconds up to a nanoseconds, the kinetic fragmentation reactions proceed on the ground state potential surfaces. Once the biradical, DT_hot_, and the FA-1 reaction intermediates are depleted, the final photoproducts consist of 35 ± 2% FA and 65 ± 2% FB (the details are discussed in the “The photoproducts” section in Supplementary Text). It is possible that further, consecutive reactions take place at longer timescales, but they would not be apparent in the scattering experiment as the molecules exit the interaction region and are no longer observable.

**Fig. 4.**
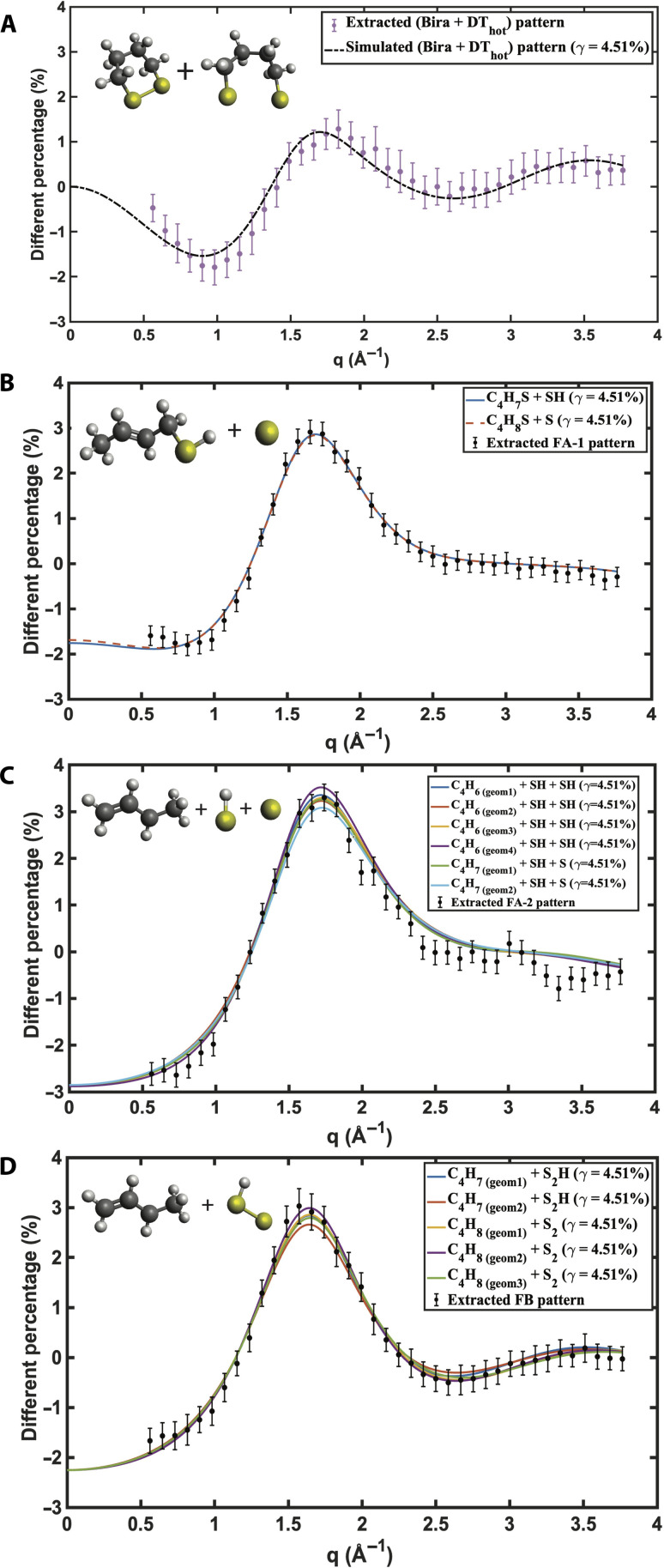
Experimental extracted (data points) and computationally modeled (solid lines) percent difference scattering patterns of reaction transients and photoproducts of the photoinduced chemical reaction of DT. The simulated scattering patterns are scaled by the optical excitation probability γ = 4.5%. (**A**) Sum of the biradical and DT_hot_. (**B**) Intermediate fragment FA-1. (**C** and **D**) End photoproducts FB and FA-2, respectively. Shown in each panel are similar fragments for various placements of the hydrogen atoms. The insets show the molecular structures used for the simulations. The error bars of the experimental scattering patterns are from multiple iterative kinetic fitting analyses.

The multidimensional nonlinear fit of the percent difference scattering maps (Fig. 2B) yields the scattering patterns for the intermediate and final species of the photochemical reaction scheme. Comparison to theoretically simulated scattering patterns (Fig. 4) reveals their identity. Only the initially prepared DT*, i.e., the electronically excited reactant, was too short-lived to resolve. The fragment species are simulated using the independent atom model (IAM) with ground-state equilibrium structures optimized at the B3LYP/6-311+*G*(*d*) level of theory. The structurally flexible species of Fig. 4B, Fig. 4C, and Fig. 4D may exist in several different conformers with different distributions and placements of the hydrogen atoms. As seen, their theoretical scattering patterns are very similar to each other, so that a further decomposition into separate conformers was not warranted. Because the fragmentation reactions likely consume a fair portion of the excitation energy, it is plausible that the species present at later times are near their respective equilibrium structures. A comparison of the experimental and modeled scattering patterns (Fig. 4) shows an excellent agreement despite of these approximations.

To ensure that the multidimensional nonlinear kinetic fit reaches the global minimum rather than a local minimum, the random initial guesses for the shared kinetic parameters and the scattering patterns for each species, i.e., $S_{\alpha }\left(q\right)$, are set to vary within a wide range. Repeating the global kinetic fittings 100 times with different initial conditions, all fits converged to a tight region with small SDs. The scattering patterns for the reaction transients shown in Fig. 4 and the reaction rates in Table 1 are the mean of the 100 trials, and corresponding error bars are the SDs. The small SDs suggest that the model is not sensitive to the initial guesses and that it reliably finds a global or near-global minimum in the optimization landscape. The low variability in the optimization results indicates that the dataset is well-suited to chosen model, meaning the model captures the underlying kinetic mechanism without being sensitive to the starting point of the optimization. The underlying source of the errors is mostly the robustness of the kinetic model and the shot noise of the experimental data.

The reaction channel leading initially to the biradical is characterized, in the early picosecond regime, by the establishment of a dynamic equilibrium between the biradical and the ${\text{DT}}_{\text{hot}}$, with forward and backward rates *k*_2_ and *k*_−2_, respectively. The optical excitation and electronic relaxation leave these transient species with 6.2 eV and 4.59 eV of internal energy for ${\text{DT}}_{\text{hot}}$ and biradical, respectively. This suggests that they are vibrationally quite hot and that structures far from the equilibrium geometries may be observed. To simulate the corresponding scattering patterns for these vibrationally hot molecules, we adopted the method introduced by Yong *et al*. (*27*) (see details in Materials and Methods). Because the biradical and the DT_hot_ species are structurally similar to the dithiane reactant, their percent difference scattering patterns have a low amplitude and, correspondingly, are measured with a low signal-to-noise ratio. Figure 4A compares the experimental scattering signal of the sum of the biradical and DT_hot_ species to the simulated one, showing satisfactory agreement. A decomposition of the experimental pattern into the individual components was not possible with the present dataset.

## DISCUSSION

**Fig. 5.**
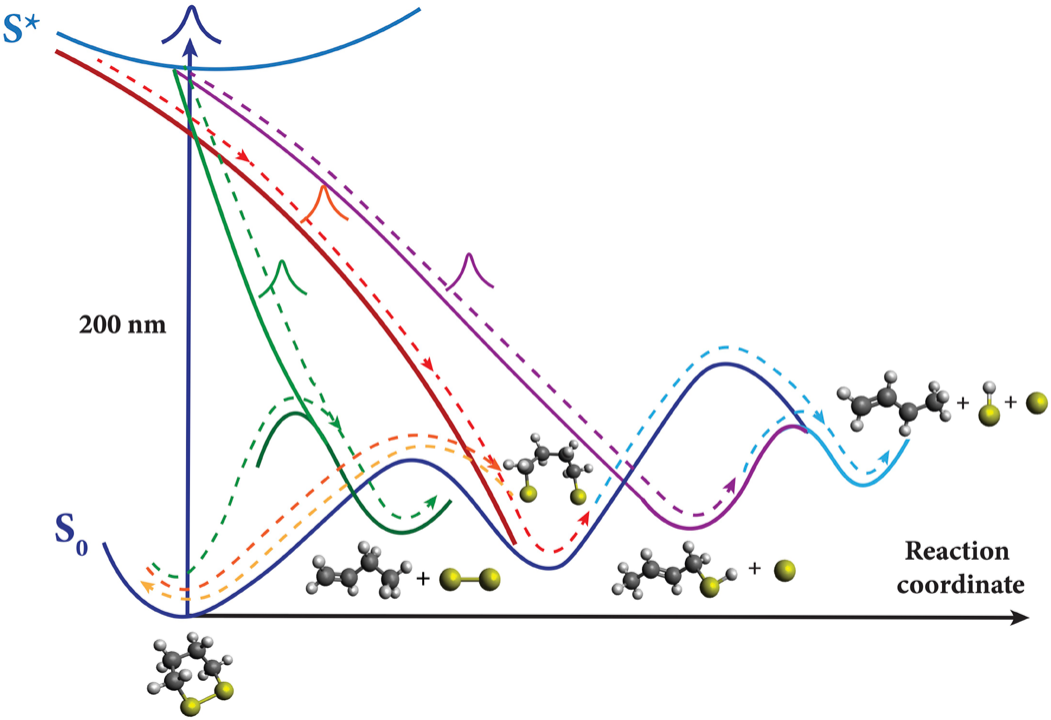
The conceptual potential energy surface and reaction scheme of DT upon excitation with 200-nm photons. One reaction pathway (red dashed line) involves the rupture of the S─S disulfide bond, leading to biradical species. Two separate, fast dissociation pathways lead to the intermediates C_4_S + S (FA-1, purple dashed line) and end photoproducts C4 + S + S and C_4_ + S_2_ (blue and green dashed lines), with a distribution of 36 and 64%, respectively. The color coding matches Fig. 3.

The ultrafast time-resolved x-ray scattering experiment of DT reveals a complex photochemical reaction network upon deep-UV excitation, shown schematically in Fig. 5. From previous studies, it is already known that optical excitation of DT near 200 nm reaches a set of high-lying electronic states that are interconnected to the ground-state surface via multiple conical intersections (*11*). We now uncover that absorption of a 200 nm photon induces several reaction pathways on sub-picosecond timescales. This includes rupture of the disulfide bond, leading to the biradical species S-C_4_-S. Other fast pathways split the molecule into C_4_H_8−*x*_S + SH_*x*=0 or 1_ (FA-1) and C_4_H_8−*z*_ + S_2_H_*z*=0 or 1_ (FB) fragments, respectively. Those same photoproducts also appear on slower timescales, so that after a nanosecond, essentially all molecules have fragmented, resulting in a distribution of 64% of two-particle fragmentation (C_4_H_8−*z*_ + S_2_H_*z*=0 or 1_) and 36% three-particle fragmentation (C_4_H_8−*x*−*y*_ + SH_*x*=0 or 1_ + SH_*y*=0 or 1_). We note that because x-ray scattering is rather insensitive to hydrogen atoms, their exact location on different fragments could not be determined. As a result, there is ambiguity regarding the energetics of the transient and product species, rendering Fig. 5 conceptual rather than quantitative.

By following the time-dependent x-ray scattering patterns of DT upon excitation, we have recorded the arrival of the molecule on the ground-state surface and a very rapid, almost explosive ejection of S and S_2_ species as a major pathway. Although we observe these nascent fragments on timescales of hundreds of femtoseconds, the time resolution of the present experiment was not sufficient to reveal the detailed wave packet dynamics of the most rapid early stages. Further investigations by harder x-rays are anticipated to provide further insights. Simulations by Kirrander *et al*. (*28*) have demonstrated that the shape of the wave packet propagating on excited-state surfaces are mirrored in the x-ray scattering patterns as intricately detailed modulations at momentum transfer vectors above 7 Å^−1^. In addition, progress has recently been made in determining the electron density distributions of electronically excited states (*29*, *30*). Experiments with better signal-to-noise ratios, harder x-rays, and better temporal resolution could be very rewarding for determining the earliest steps of the fascinating photochemical reaction of DT.

## MATERIALS AND METHODS

### Experimental methods

The time-resolved gas-phase x-ray scattering measurements were carried out with at the coherent x-ray imaging instrument (*31*) at the LCLS (*12*) at SLAC National Accelerator Laboratory. The 200-nm optical pump laser was generated from the fourth harmonic of a 120-Hz Ti:Sapphire laser amplifier with an ~80-fs pulse duration, with ~1 μJ per pulse on target, and a ~1.5-nm spectral bandwidth. The x-ray probe pulse generated from LCLS operated at 120 Hz with ~10^12^ photons per pulse at 9.5-keV photon energy with a 20-eV full width at half maximum bandwidth and a ~30-fs pulse duration. The laser focus, about 50 μm, was slightly larger than the focus of the x-ray beam, approximately 30 μm. The cross-correlation time of the pump and probe pulses was determined to be 238 ± 22 fs from the onset of the observed time-dependent scattering signals. The gaseous DT sample pressure was controlled by a piezoelectric needle valve to ~2 torr of pressure in the interaction region. The gas cell and the CSPAD detector are both in vacuum, with an average background pressure outside of the scattering cell of 1.7 × 10^−5^ torr, which is mostly comprised of the DT that flows out of the windowless scattering cell. The pulse energy and gas pressure were optimized for a reduced background signal and a <5% excitation probability. The interaction length was kept small, 2.4 mm, to prevent excessive attenuation of the UV beam by the sample in the interaction region.

The pump-probe delay time was controlled by a motorized delay stage, and the shot-to-shot timing jitter of the x-ray beam was monitored with a specialized timing tool (*32*). The actual time delay of each shot was then determined to be the sum of the laser stage position and the edge position of the time tool. Furthermore, the shot-to-shot x-ray pulse energy was monitored by a photodiode downstream of the scattering cell. To achieve the necessary noise level (<0.1%), it was necessary to calibrate the intensity after the scattering cell because the x-ray also has spatial jitter that affects the transmission of the x-ray through the Pt pinholes (see Fig.1). The scattered x-rays were detected via a 2.3-megapixel CSPAD (*20*). Details of the detector calibration, the error analysis of the measured scattering signals, and the decomposition into isotropic and anisotropic signals are found in (*18*, *26*).

Dithiane was synthesized and isolated as described in ref. (*8*). The starting material is 1,4-butanedithiol. The purity was verified in nuclear magnetic resonance and gas chromatography–mass spectrometry experiments.

### Computational methods

To calculate the percent difference scattering signals of FA-1 and FB, we optimize their ground-state equilibrium structures at the B3LYP/6-311+*G*(*d*) level of theory and simulate scattering patterns using the IAM. The IAM treats the molecular electron density as a sum of the spherical atomic densities, each centered at the position of the respective nuclei. It neglects the formation of chemical bonds, the polarization of atoms in a molecule, and the electronic excitations. While an accurate description of the electron density is important to determine the molecular structure with very high accuracy (*29*, *33*), IAM is a good option for identifying the heavy-atom structure of the transient intermediates and final photoproducts, especially when intricate fragmentation patterns occur in photochemical reactions. The fission of the DT* molecules consumes a fair amount of the excitation energy, so that it is likely that the species FA-1 and FB, present at later times, are near their respective equilibrium structures. Considering that both FA-1 and FB are on their ground electronic surfaces and are relatively stable compared to other transient intermediates, the IAM is sufficient to simulate those scattering patterns (*34*).

The ground-state dynamics of vibrationally hot DT and hot biradicals are simulated using surface hopping including arbitrary couplings (SHARC) (*35*) interfaced with the electronic structure package MOLPRO (*36*). A total of 12,832 geometries calculated from molecular dynamics (MD) are extracted to represent an ensemble of vibrationally hot DT at the thermal equilibrium, while 54,505 geometries are used for the hot biradical. Molecular dynamics is used to generate expansive pools of physically reasonable structures of vibrationally hot DT and biradicals. These structure pools are then used to model the x-ray scattering patterns of the vibrationally hot ensemble. By varying the energy input, the internal energy of the system is adjustable and, in this case, is chosen to match the 200-nm laser photon energy, i.e., 6.2 eV of excess kinetic energy. The initial conditions for hot DT are sampled from Wigner distributions to account for the zero-point vibrations. The 6.2 eV of excess kinetic energy was randomly distributed among all degrees of freedom of the molecules. The electronic structure calculations during the molecular dynamics are performed at the complete active space self-consistent field (CASSCF)(8,10)/6-31G(d,p) level. Similarly, the initial conditions for the hot biradical are also sampled from Wigner distributions, and the relative equilibrium energy of biradical (singlet, 1.610 eV), relative to DT, is obtained at the same level of theory. Different degenerate states are not an issue for this calculation, because the singlet state is the lowest energy state for biradical. The reaction leading to the biradical via singlet states has been discussed previously by Rankine et al. (*10*) and Larsen et al. (*11*). Thus, for the 6.2 eV of photon energy, the total excess kinetic energy for the biradical is 4.59 eV either way. A total of 40 trajectories are calculated for DT up to 3 ps, while a total of 49 trajectories are calculated for biradical up to 3 ps. The 0.5-fs time steps are chosen to ensure that the energy is conserved during the MD calculations while keeping the computational time reasonable. After obtaining the structure pools of hot DT and biradical, we simulate the scattering patterns following the method introduced by Yong *et al.* (*27*).
