# Supplementary material for: Revealing the reaction path of UVC bond rupture in cyclic disulfides with ultrafast x-ray scattering
Source: Sci Adv. 2025 Jan 15;11(3):eadp9175. doi: 10.1126/sciadv.adp9175 (PMC11734709; doi:10.1126/sciadv.adp9175)
Supplement: Supplementary file 1 — Supplementary Text Figs. S1 to S11 Tables S1 to S11 [file sciadv.adp9175_sm.pdf]

Science Advances
AAAS

Supplementary Materials for

Revealing the reaction path of UVC bond rupture in cyclic disulfides with ultrafast x-ray scattering

Lingyu Ma *et al.*

Corresponding author: Peter M. Weber, peter_weber@brown.edu

Sci. Adv. **11**, eadp9175 (2025)
DOI: 10.1126/sciadv.adp9175

This PDF file includes:

Supplementary Text
Figs. S1 to S11
Tables S1 to S11

Supplementary Text

1. Additional details about the kinetics fit

Because the kinetics scheme (see Fig. 3b) for the photochemical reaction of DT upon 200 nm excitation is complicated, it is impossible to directly solve the differential rate equations analytically. We therefore separated the overall kinetics into different time domains that were separately solved.

As seen in Fig. 2a, the 200 nm photons pump the DT molecules into an excited state that reacts in the femtosecond time domain. Scattering patterns in the early picosecond time domain, i.e. the patterns between 1 ps and 15 ps, show a transient phase that involves the thermalization of the energy and the establishment of a fast equilibrium of structural interconversion between biradical and DT_{hot}, revealing the initial photochemical reaction transients. At the longer time delays, from 15 ps to 3 ns, there are further changes in the percent difference scattering signals. This time range is quite typical for the consecutive kinetic reactions of the reaction intermediates generated at earlier times. We therefore separate the overall experimental percent difference scattering signals into two main time domains to perform the kinetic analysis: the short-time data covering the femtosecond and early picosecond time scale, up to 15 ps, and the long-time signals from 15 ps up to 3 ns.

We use the kinetic model shown in Scheme S1 to describe the early photochemical reactions underlying the short-time experimental percent difference scattering data.

Scheme. S1 | Kinetics Model to Analyze Short-time Experimental Data

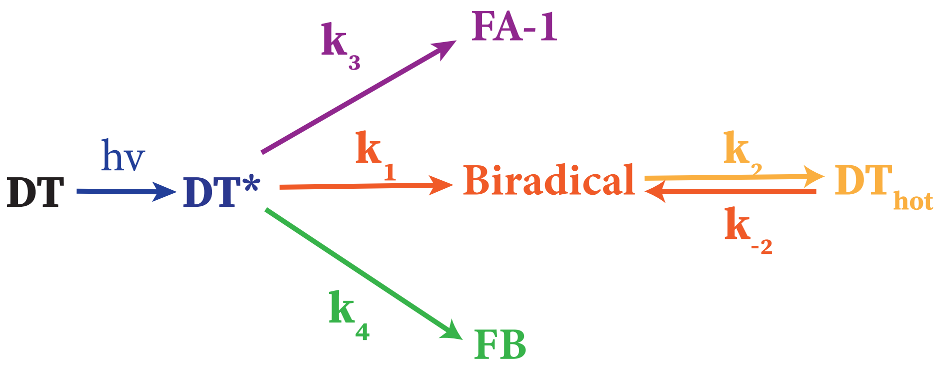

Assuming first order kinetics, the system follows the differential rate equations

$$\frac{d[DT^*]}{dt} = -(k_1 + k_3 + k_4) \cdot [DT^*]$$

$$\frac{d[Bira]}{dt} = k_1[DT^*] + k_{-2}[DT_{hot}] - k_2[Bira]$$

$$\frac{d[DT_{hot}]}{dt} = k_2[Bira] - k_{-2}[DT_{hot}]$$

$$\frac{d[FA-1]}{dt} = k_3[DT^*]$$

$$\frac{d[FB]}{dt} = k_4[DT^*] \qquad (S1)$$

Here, $[DT^*]$, $[Bira]$, $[DT_{hot}]$, $[FA-1]$ and $[FB]$ are the relative populations of electronically excited DT, Biradical, vibrationally hot DT, FA-1 and FB species, respectively. k_1, k_3 and k_4 represent rate constants for the initially excited DT molecules decaying from those high-lying excited states to form corresponding species. The k_2 and k_{-2} rate constants describe the fast equilibrium of structural interconversion between Biradical and DT_{hot}. We solve the Eqs. (S1) with the initial conditions:

$$[DT^*](t_0) = A_0$$

$$[Bira](t_0) = [DT_{hot}](t_0) = [FA-1](t_0) = [FB](t_0) = 0$$

Here, t_0 is the time when excited DT molecules decay from the excited state. According to the photoelectron study by *Larsen et al.* (*11*). the decay of S_5 and the rise of S_{5v} and S_2 is around 44 ± 4 fs. Thus, t_0 is estimated as 50 fs for the kinetic fits and the corresponding $A_0 = 1$.

We derive the following time-dependent functions of the relative population for species:

$$[DT^*](t) = A_0 \cdot e^{-(k_1+k_3+k_4)\cdot(t-t_0)}$$

$$[Bira](t) = A_0 \cdot ((A_{11} - A_{12}) \cdot e^{-(k_1+k_3+k_4)\cdot(t-t_0)} - A_{11} \cdot e^{-(k_2+k_{-2})\cdot(t-t_0)} + A_{12})$$

$$[DT_{hot}](t) = A_0 \cdot (-A_{21} \cdot e^{-(k_1+k_3+k_4)\cdot(t-t_0)} + A_{11} \cdot e^{-(k_2+k_{-2})\cdot(t-t_0)} + C_1)$$

$$[FA-1](t) = A_0 \cdot (-A_{31} \cdot e^{-(k_1+k_3+k_4)\cdot(t-t_0)} + A_{31})$$

$$[FB](t) = A_0 \cdot \left(-A_{41} \cdot e^{-(k_1+k_3+k_4)\cdot(t-t_0)} + A_{41}\right) \qquad (S2)$$

Where:

$$A_{11} = \frac{k_1 \cdot k_2}{(k_2+k_{-2})\cdot(-k_1+k_2+k_{-2}-k_3-k_4)} \qquad A_{12} = \frac{k_1 \cdot k_{-2}}{(k_2+k_{-2})\cdot(k_1+k_3+k_4)}$$

$$A_{21} = \frac{k_1 \cdot k_2}{(k_1+k_3+k_4)\cdot(-k_1+k_2+k_{-2}-k_3-k_4)} \qquad A_{31} = \frac{k_3}{k_1+k_3+k_4}$$

$$A_{41} = \frac{k_4}{k_1+k_3+k_4} \qquad C_1 = \frac{k_1 \cdot k_2}{(k_2+k_{-2})\cdot(k_1+k_3+k_4)}$$

According to Eq. (2), the isotropic percent difference scattering signal is represented as:

$$\Delta I_{iso}(q,t) = \gamma \left(S_{DT^*}(q) \cdot [DT^*](t) + S_{Bira}(q) \cdot [Bira](t) + S_{DT_{hot}}(q) \cdot [DT_{hot}](t) + S_{FA-1}(q) \cdot [FA-1](t) + S_{FB}(q) \cdot [FB](t)\right) \cdot H(t \geq t_0) \qquad (S3)$$

The $H(t > t_0)$ is a Heaviside step-function. The γ is the fraction of molecules that are optically excited, a scalar quantity that is intended to be kept low in order to minimize multi-photon excitation processes (*26*). The time-independent percent difference scattering pattern of DT*, Biradical, DT_{hot}, FA-1 and FB are represented by $S_{DT^*}(q)$, $S_{Bira}(q)$, $S_{DT_{hot}}(q)$, $S_{FA-1}(q)$ and $S_{FB}(q)$, respectively, with adjustable q dependence. Each $S_x(q)$ is parameterized by a set of points in momentum transfer domain with x being DT*, biradical, DT_{hot}, FA-1, and FB. The time-dependence arises from the relative populations for the respective species as described by the kinetics scheme shown in Scheme. S1. Therefore, we simplify Eqs (S3) into a compact format:

$$f(q,t) = \{A_0 \cdot \gamma \cdot (D_1(q) \cdot e^{-(k_1+k_3+k_4)\cdot(t-t_0)} + D_2(q) \cdot e^{-(k_2+k_{-2})\cdot(t-t_0)} + D_3(q))\} \cdot H(t \geq t_0) \qquad \text{(S4)}$$

with

$$D_1(q) = S_{DT^*}(q) + S_{Bira}(q) \cdot (A_{11} - A_{12}) - S_{DT_{hot}}(q) \cdot A_{21} - S_{FA-1}(q) \cdot A_{31} - S_{FB}(q) \cdot A_{41}$$

$$D_2(q) = (S_{DT_{hot}}(q) - S_{Bira}(q)) \cdot A_{11}$$

$$D_3(q) = S_{Bira}(q) \cdot A_{12} + S_{DT_{hot}}(q) \cdot C_1 + S_{FA-1}(q) \cdot A_{31} + S_{FB}(q) \cdot A_{41}$$

We note that although the kinetic model cannot fully describe the dynamical aspects of the reaction at very early times (less than about 200 fs), the analysis appears to work quite well for all but the very early time points. This also indicates that the energy actually thermalizes sufficiently quickly for the later parts of the kinetic analysis to hold. The kinetics fits for the short-time experimental percent difference scattering data yields the scattering patterns for biradical + DT_{hot}, FA-1 and FB as shown in Fig. 4, respectively, and also the reaction rates k_1, k_2, k_{-2}, k_3 and k_4 and their corresponding time constants as shown in Table 1.

As for the origins of error bars of the scattering patterns for species shown in Fig. 4 and the reaction rates shown in Table 1, every time we perform a global kinetic fit on short-time experimental data, it yields a set of fit results including shared kinetic parameters and scattering patterns for each species. Those values fluctuate because the global kinetic fits involve a random initial guess. Thus, in order to minimize any bias introduced by the random initial guess, we repeat the global kinetic fits until convergence is reached. We find that 100 runs are sufficient. The final fit result is the mean of the 100 individual fits, and the corresponding error bars are the standard deviation associated with the 100 fits.

In order to fully describe the trends of the long-time experimental percent difference scattering signals, we use the kinetics model shown in Scheme S2.

Scheme S2 | Kinetics Model to Analyze Long-time Experimental Data

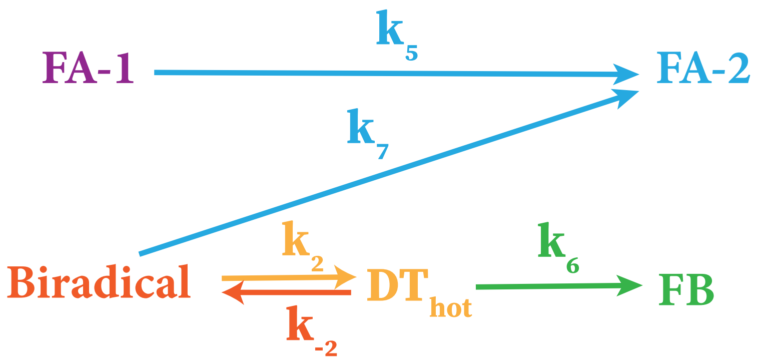

Again, assuming first order kinetics, the system is described by the following set of differential rate equations:

$$\frac{d[Bira]}{dt} = k_{-2}[DT_{hot}] - (k_2 + k_7)[Bira]$$

$$\frac{d[DT_{hot}]}{dt} = k_2[Bira] - (k_{-2} + k_6)[DT_{hot}]$$

$$\frac{d[FA-1]}{dt} = -k_5[FA-1]$$
$$\frac{d[FA-2]}{dt} = k_5[FA-1] + k_7[Bira]$$
$$\frac{d[FB]}{dt} = k_6[DT_{hot}] \qquad \text{(S5)}$$

Here, [FA-2] is the relative population of FA-2. The k_5, k_6 and k_7 are rate constants for consecutive kinetic reactions involving the dissociations. The starting conditions for the long-time experimental fits should be consistent with the results of the short-time fits at the end time points. Thus,

$$[Bira](t = 15\,ps) = 0.102$$
$$[DT_{hot}](t = 15\,ps) = 0.310$$
$$[FA-1](t = 15\,ps) = 0.261$$
$$[FA-2](t = 15\,ps) = 0.031$$
$$[FB](t = 15\,ps) = 0.297$$

Through the analysis of the possible structural pairs of photoproducts at the very end time points of the experimental time window, i.e. 700 ps and 1 ns (see below for details), we also get boundary conditions:

$$[FB](t = t_{final}) = 0.648 \pm 0.023$$
$$[FA-2](t = t_{final}) = 0.352 \pm 0.023$$

The t_{final} refers to the time point when the relative population curves flatten out. Similarly, the long-time isotropic percent difference scattering signal is represented as:

$$\Delta I_{iso}(q,t) = \gamma\Big(S_{Bira}(q)\cdot[Bira](t) + S_{DT_{hot}}(q)\cdot[DT_{hot}](t) + S_{FA-1}(q)\cdot[FA-1](t) + S_{FA-2}(q)\cdot[FA-2](t) + S_{FB}(q)\cdot[FB](t)\Big)\cdot H(t \geq t_1) \qquad (S6)$$

with $t_1 = 15\,ps$, the starting point for the long-time kinetics fits. Since the precent excitation, γ, is already known, k_2, k_{-2}, $S_{Bira}(q)$, $S_{DT_{hot}}(q)$, $S_{FA-1}(q)$, $S_{FB}(q)$ from the kinetics fits of short-time experimental data, the scattering signal of FA-2 ($S_{FA-2}(q)$) and the remaining unknow kinetics parameters k_5, k_6, and k_7 are treated as adjustable parameters in the long-time experimental signal fits. The time-dependent relative population of each species, i.e.: $[Bira](t)$, $[DT_{hot}](t)$, $[FA-1](t)$, $[FA-2](t)$ and $[FB](t)$, are obtained analytically by solving differential equations A5 with boundary conditions. A fit to the long-time experimental signals with adjustable parameters, $S_{FA-2}(q)$, k_5, k_6 and k_7 lead to the experimental scattering pattern of FA-2 presented in Fig. 4d and the kinetics parameters shown in Table 1. The simulated percent difference scattering signals are good agreement with the experimental data as indicated by the small residual shown in Fig. 2c. The error bars are derived as described in the kinetics fits of the short-time experimental signals.

Combining the fit results of the short-time and long-time experimental percent difference scattering signals, we obtain the values of all rate constants involved in the kinetics scheme shown in Fig. 3b, making it possible to solve the differential rate equations numerically. Based on the scheme in Fig. 3b, the overall photoinduced chemical reaction can be represented by the following set of differential rate equations:

$$\frac{d[DT^*]}{dt} = -(k_3 + k_1 + k_4)[DT^*]$$
$$\frac{d[Bira]}{dt} = k_1[DT^*] + k_{-2}[DT_{hot}] - (k_2 + k_7)[Bira]$$

$$\frac{d[DT_{hot}]}{dt} = k_2[Bira] - (k_{-2} + k_6)[DT_{hot}]$$
$$\frac{d[FA-1]}{dt} = k_3[DT^*] - k_5[FA-1]$$
$$\frac{d[FB]}{dt} = k_4[DT^*] + k_6[DT_{hot}]$$
$$\frac{d[FA-2]}{dt} = k_5[FA-1] + k_7[Bira] \qquad \text{(S7)}$$

with the initial conditions:

$$[DT^*](t_0) = 1,\ [Bira](t_0) = 0,\ [DT_{hot}](t_0) = 0$$
$$[FA-1](t_0) = 0,\ [FA-2](t_0) = 0,\ [FB](t_0) = 0$$

and the reaction rates shown in Table 1 as determined in the separate fits. Plotting the time-dependent numerical solutions of different species against the pump-probe delay, we obtain the corresponding time-involving relative population of transients shown in Fig. 3b.

By using the reaction rates and extracted scattering patterns, the simulated percent difference scattering signals are generated (Fig. 2b) and the residuals can be calculated (Fig. 2c). To examine the consistency between the experimental patterns and the simulated ones more carefully and to show the quality of the kinetics fit, Fig. 2d shows a direct comparison between the experimental and simulated patterns at representative delay times. It is apparent that all of them give excellent fits within the whole q range at all selected time points, which supports the proposed kinetic model.

2. The photoproducts

In order to determine the photoproducts and the boundary conditions for the kinetic fits of the long-time experimental percent difference scattering data, it is necessary to fit the representative end time points, i.e. 700 ps, 1000 ps and 3000 ps. Fig. S1 shows the experimental pump-probe X-ray scattering percent difference signals at given delay times during the overall photochemical reaction of DT. It is apparent that the scattering patterns at 700 ps and 1000 ps are nearly overlapped, and the one at 3000 ps is close to them but has slightly lower scattering signals both at low and high *q*, only near the maximum are the data similar. It is most likely that the scattering signal at this time point is affected by molecules leaving the interaction region. Thus, we disregard the 3000 ps time point in the subsequent analysis. Considering the small differences between 700 ps and 1000 ps on scattering signals, it is reasonable to select either of the scattering patterns at 700 ps or 1000 ps as the representative end-time scattering signal for the trial fits.

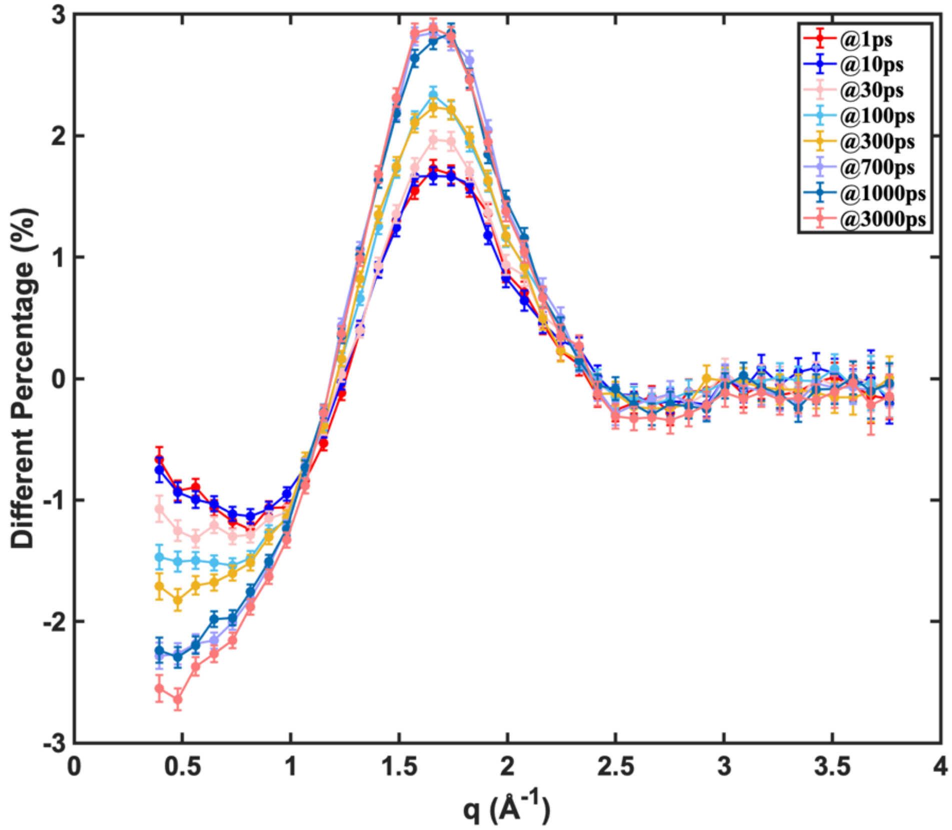

Figure S1 | Experimental pump-probe X-ray scattering percent difference signals at given representative delay times during the overall photochemical reaction of DT.

2.1 The fit results at 700 ps

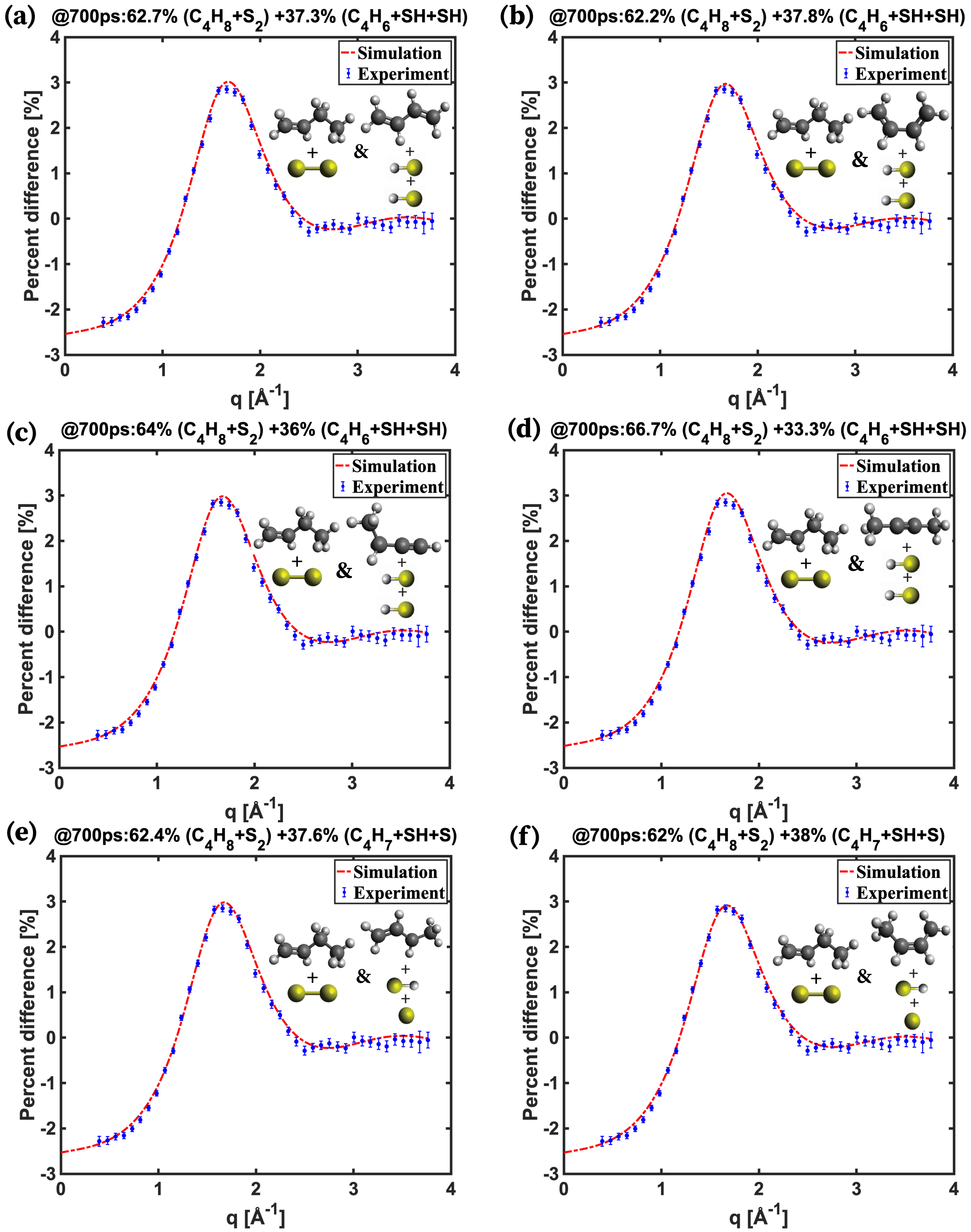

Figure S2 | The fits for the possible structure combinations of $C_4H_8\,(geom1) + S_2$ (FB) and all possible FA-2, (a-f), at 700 ps. The ratios of FA-2 and FB are all shown on the top of each subplot and obtained through least-square fits between experimental pattern and simulated patterns with percent excitation $\gamma = 4.51\%$.

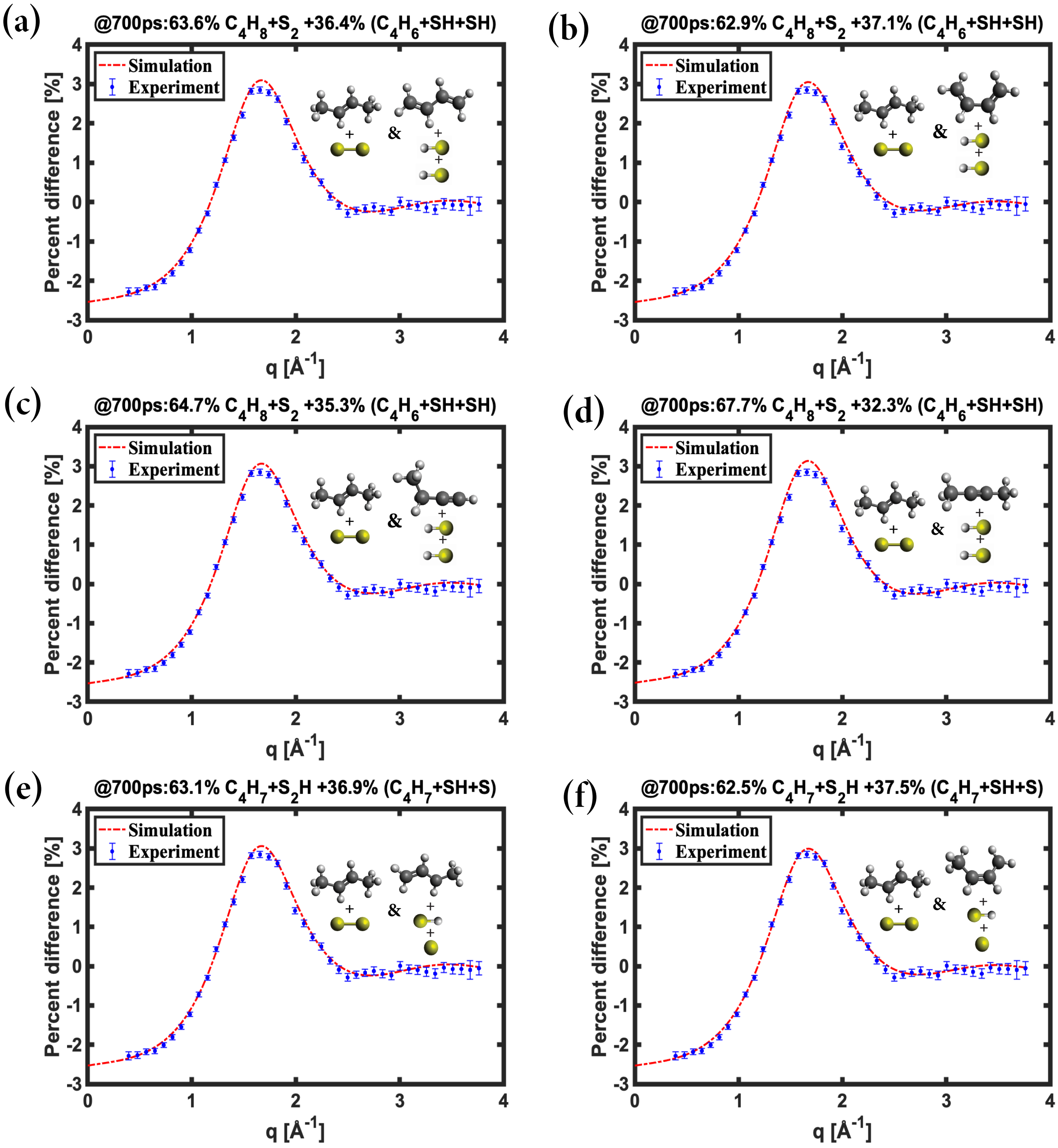

Figure S3 | The fits for the possible structure combinations of $C_4H_8\,(geom2) + S_2$ (FB) and all possible FA-2, (a-f), at 700 ps. The ratios of FA-2 and FB are all shown on the top of each subplot and obtained through least-square fits between experimental pattern and simulated one with percent excitation $\gamma = 4.51\%$.

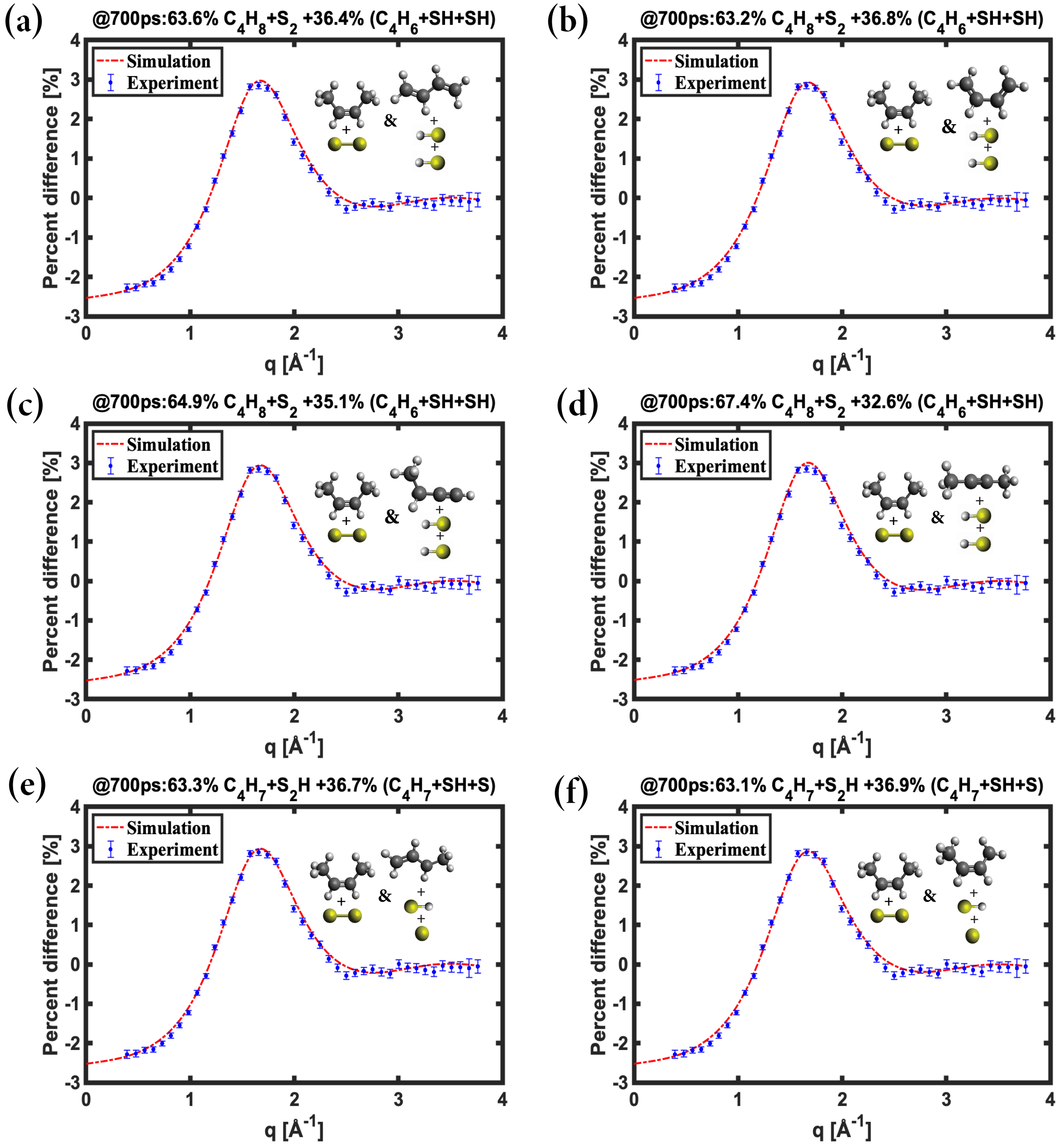

Figure S4 | The fits for the possible structure combinations of $C_4H_8\,(geom3) + S_2$ (FB) and all possible FA-2, (a-f), at 700 ps. The ratios of FA-2 and FB are all shown on the top of each subplot and obtained through least-square fits between experimental pattern and simulated one with percent excitation $\gamma = 4.51\%$.

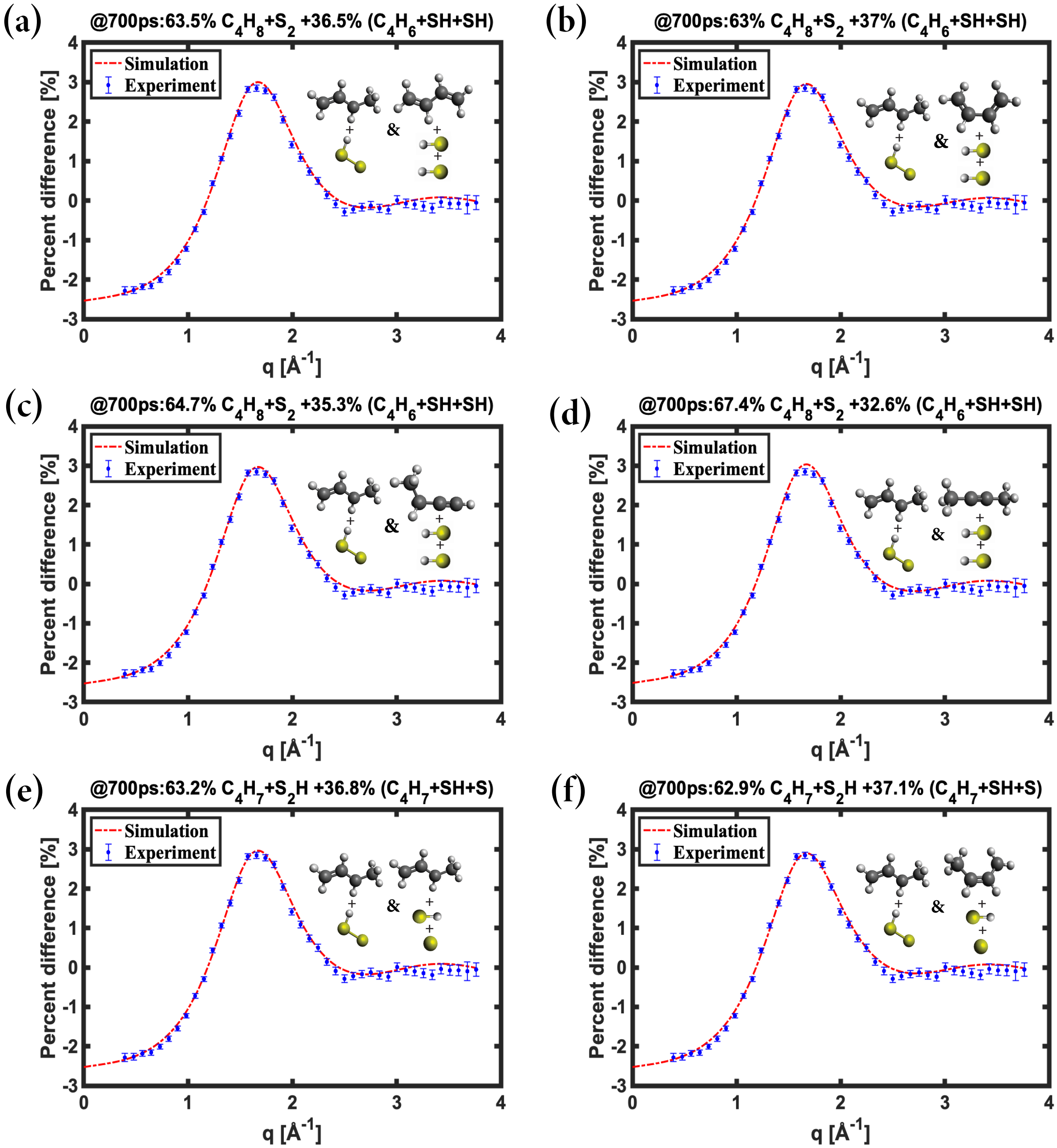

Figure S5 | The fits for the possible structure combinations of C_4H_7 $(geom1) + S_2H$ (FB) and all possible FA-2, (a-f), at 700 ps. The ratios of FA-2 and FB are all shown on the top of each subplot and obtained through least-square fits between experimental pattern and simulated one with percent excitation $\gamma = 4.51\%$.

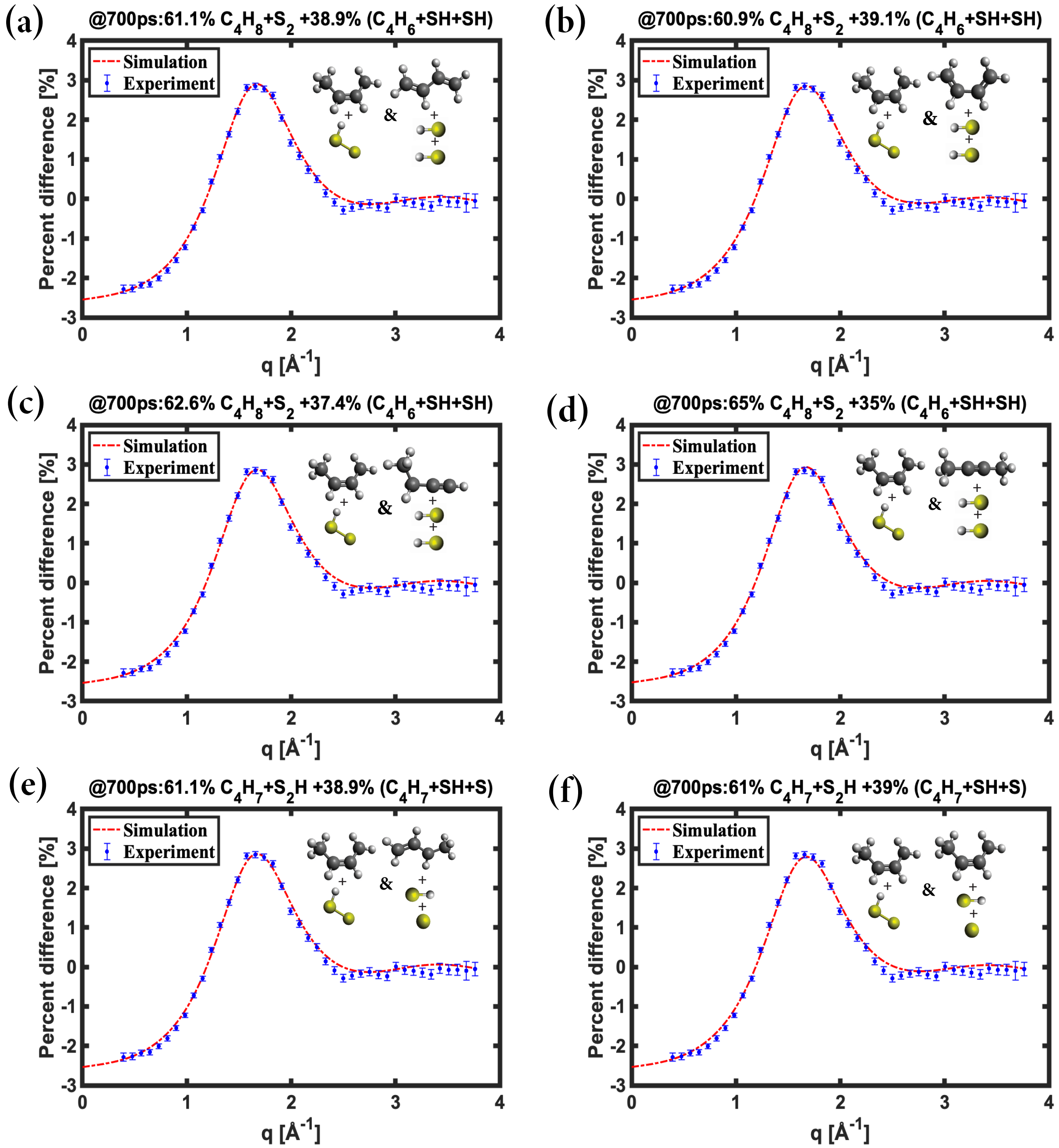

Figure S6 | The fits for the possible structure combinations of $C_4H_7\ (geom2) + S_2H$ (FB) and all possible FA-2, (a-f), at 700 ps. The ratios of FA-2 and FB are all shown on the top of each subplot and obtained through least-square fits between experimental pattern and simulated one with percent excitation $\gamma = 4.51\%$.

Table S1 | Summary of least-square fit results for all possible structural pairs of final photoproducts at 700 ps.

No.	γ (%)	**Ratio @ 700 ps**			
1	4.51	C4H8 (geom1) + S2	62.70	C4H6 (geom1, trans) + SH + SH	37.30
2	4.51		62.20	C4H6 (geom1, cis) + SH + SH	37.80
3	4.51		64.00	C4H6 (geom2) + SH + SH	36.00
4	4.51		66.70	C4H6 (geom3) + SH + SH	33.30
5	4.51		62.40	C4H7 (geom1) + SH + S	37.60
6	4.51		62.00	C4H7 (geom2) + SH + S	38.00
7	4.51	C4H8 (geom2) + S2	63.55	C4H6 (geom1, trans) + SH + SH	36.45
8	4.51		62.86	C4H6 (geom1, cis) + SH + SH	37.14
9	4.51		64.65	C4H6 (geom2) + SH + SH	35.35
10	4.51		67.68	C4H6 (geom3) + SH + SH	32.32
11	4.51		63.09	C4H7 (geom1) + SH + S	36.91
12	4.51		62.47	C4H7 (geom2) + SH + S	37.53
13	4.51	C4H8 (geom3) + S2	63.61	C4H6 (geom1, trans) + SH + SH	36.39
14	4.51		63.21	C4H6 (geom1, cis) + SH + SH	36.79
15	4.51		64.92	C4H6 (geom2) + SH + SH	35.08
16	4.51		67.45	C4H6 (geom3) + SH + SH	32.55
17	4.51		63.33	C4H7 (geom1) + SH + S	36.67
18	4.51		63.10	C4H7 (geom2) + SH + S	36.90
19	4.51	C4H7 (geom1) + S2H	63.49	C4H6 (geom1, trans) + SH + SH	36.51
20	4.51		63.02	C4H6 (geom1, cis) + SH + SH	36.98
21	4.51		64.73	C4H6 (geom2) + SH + SH	35.27
22	4.51		67.37	C4H6 (geom3) + SH + SH	32.63
23	4.51		63.22	C4H7 (geom1) + SH + S	36.78
24	4.51		62.89	C4H7 (geom2) + SH + S	37.11
25	4.51	C4H7 (geom2) + S2H	61.14	C4H6 (geom1, trans) + SH + SH	38.86
26	4.51		60.85	C4H6 (geom1, cis) + SH + SH	39.15
27	4.51		62.60	C4H6 (geom2) + SH + SH	37.40
28	4.51		65.02	C4H6 (geom3) + SH + SH	34.98
29	4.51		61.05	C4H7 (geom1) + SH + S	38.95
30	4.51		60.95	C4H7 (geom2) + SH + S	39.05
Average			63.57		36.43
STDEV			1.86		1.86

2.2 The fit results @ 1000 ps

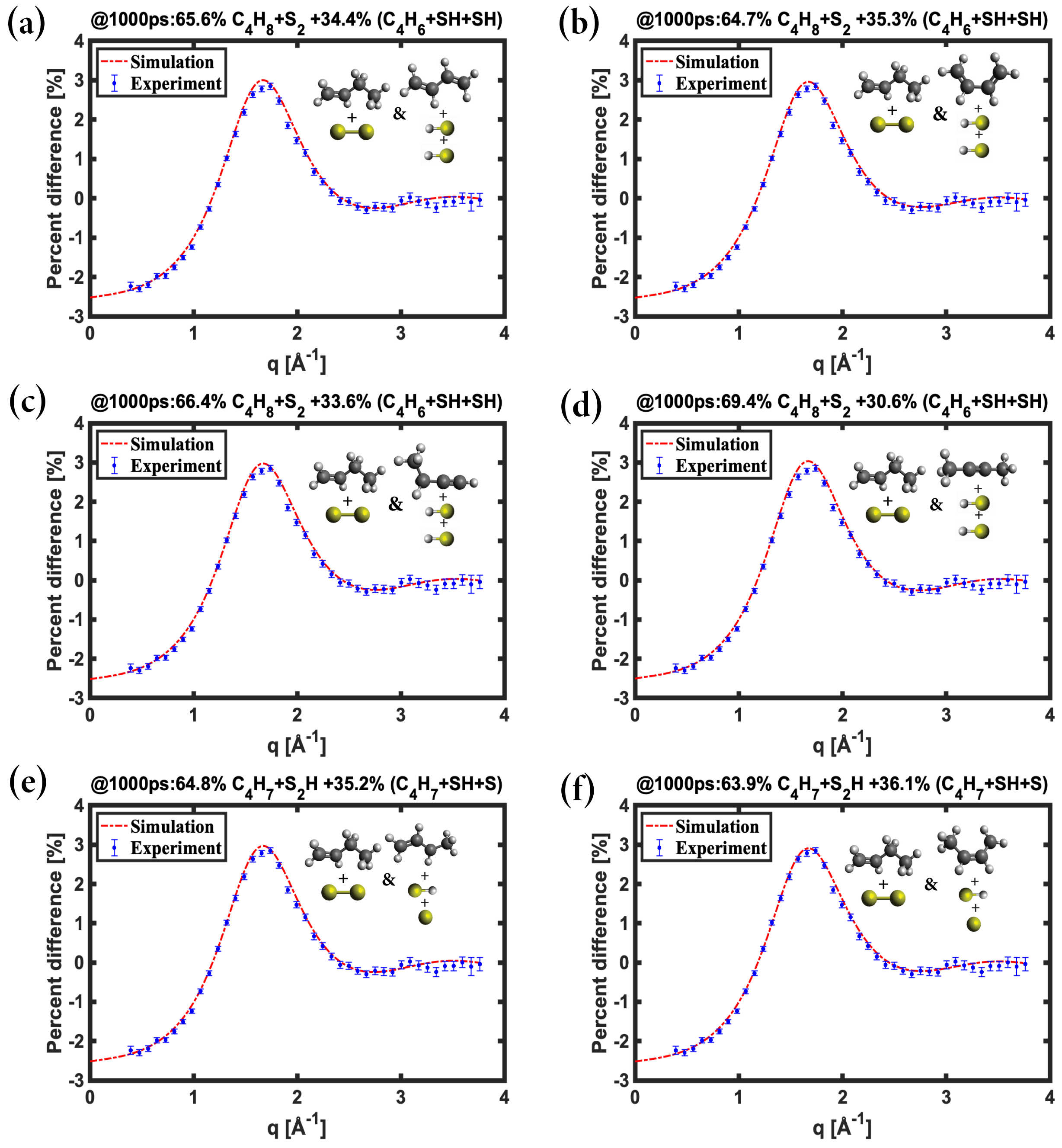

Figure S7 | The fit trials for the possible structure combinations of $C_4H_8\,(geom1) + S_2$ (FB) and all possible FA-2, (a-f), at 1000 ps. The ratios of FA-2 and FB are all shown on the top of each subplot and obtained through least-square fits between experimental pattern and simulated one with percent excitation $\gamma = 4.51\%$.

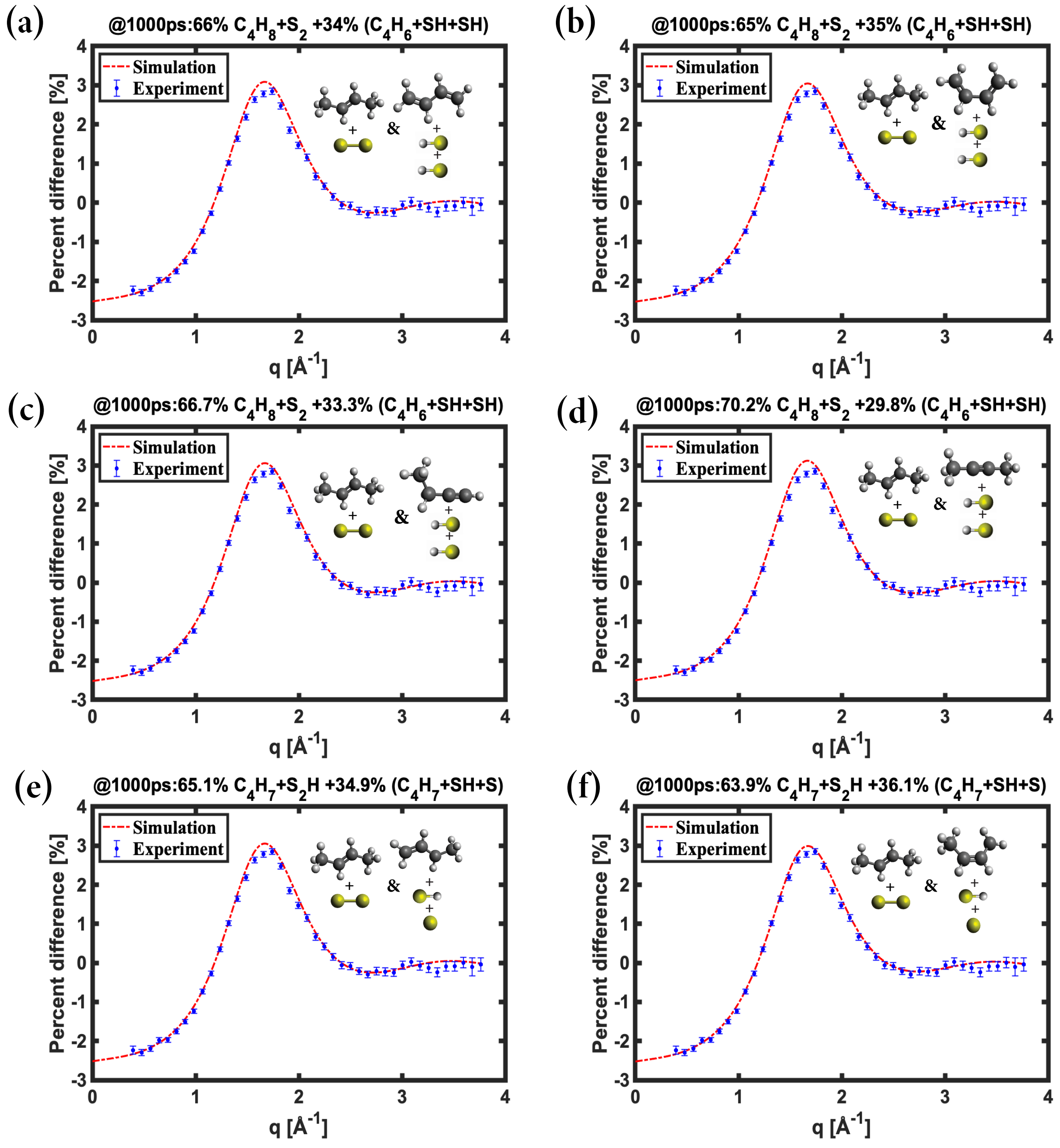

Figure S8 | The fits for the possible structure combinations of $C_4H_8\,(geom2) + S_2$ (FB) and all possible FA-2, (a-f), at 1000 ps. The ratios of FA-2 and FB are all shown on the top of each subplot and obtained through least-square fits between experimental pattern and simulated one with percent excitation $\gamma = 4.51\%$.

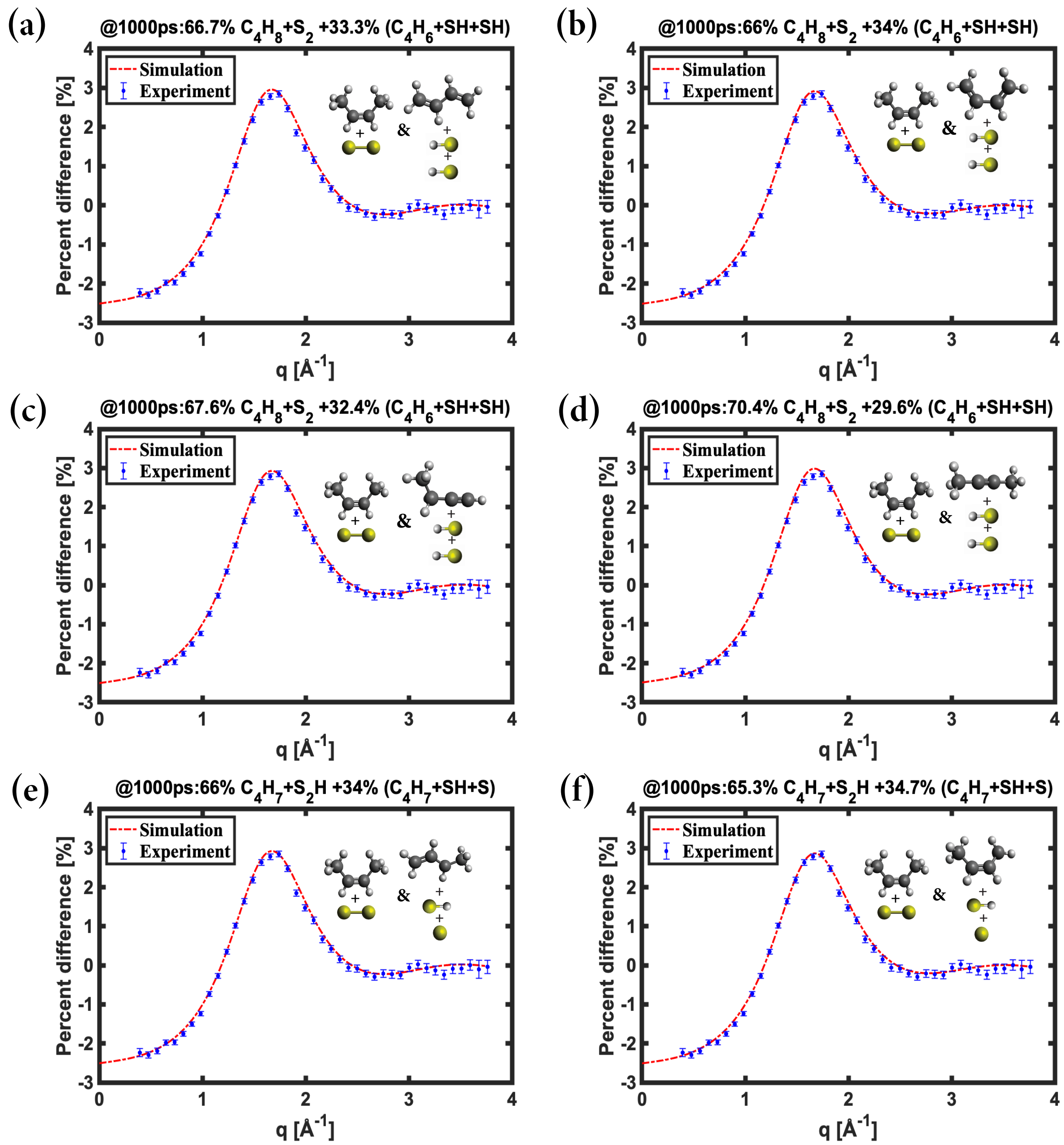

Figure S9 | The fits for the possible structure combinations of $C_4H_8\,(geom3) + S_2$ (FB) and all possible FA-2, (a-f), at 1000 ps. The ratios of FA-2 and FB are all shown on the top of each subplot and obtained through least-square fits between experimental pattern and simulated one with percent excitation $\gamma = 4.51\%$.

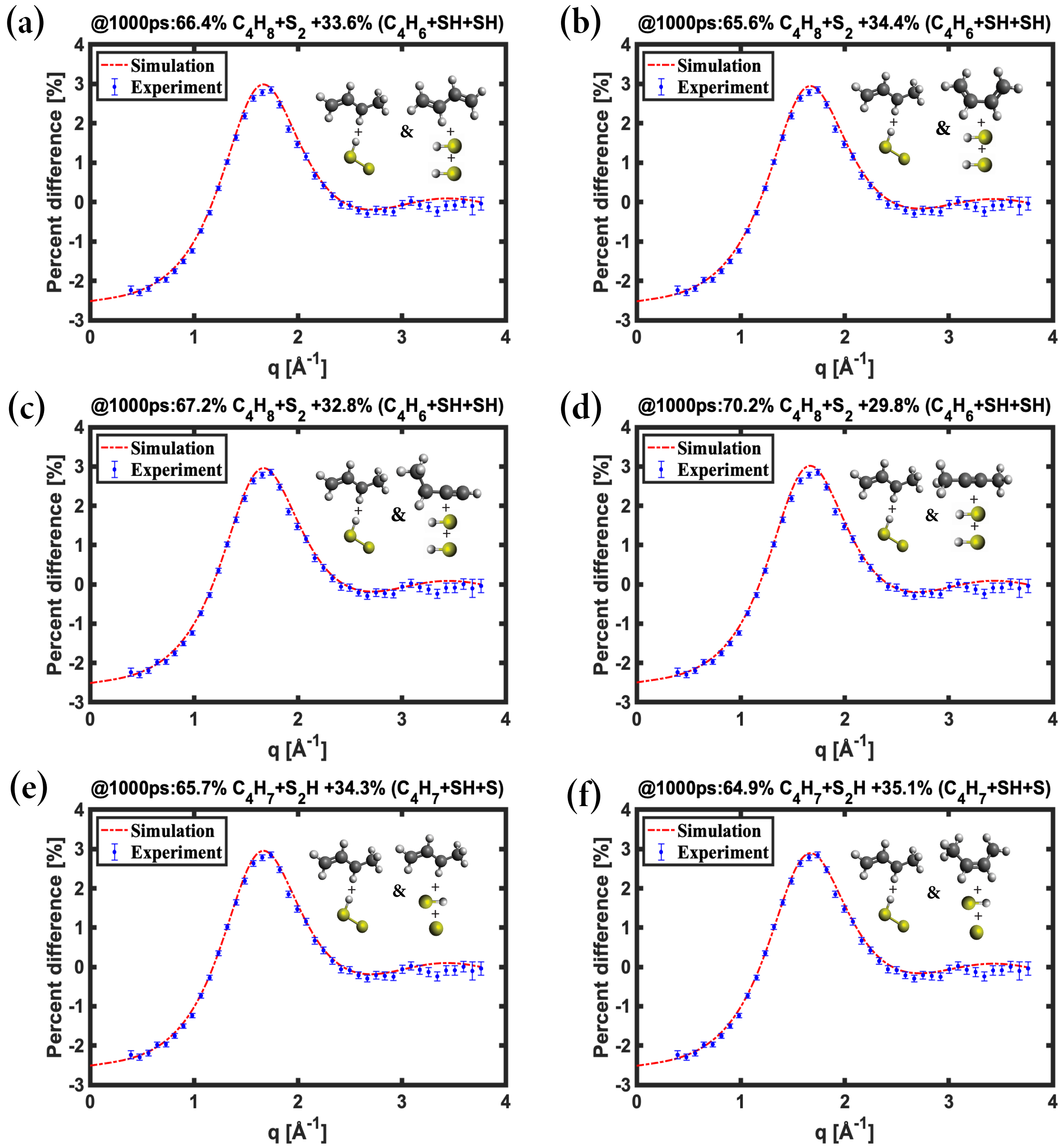

Figure S10 | The fits for the possible structure combinations of C_4H_7 $(geom1) + S_2H$ (FB) and all possible FA-2, (a-f), at 1000 ps. The ratios of FA-2 and FB are all shown on the top of each subplot and obtained through least-square fits between experimental pattern and simulated one with percent excitation $\gamma = 4.51\%$.

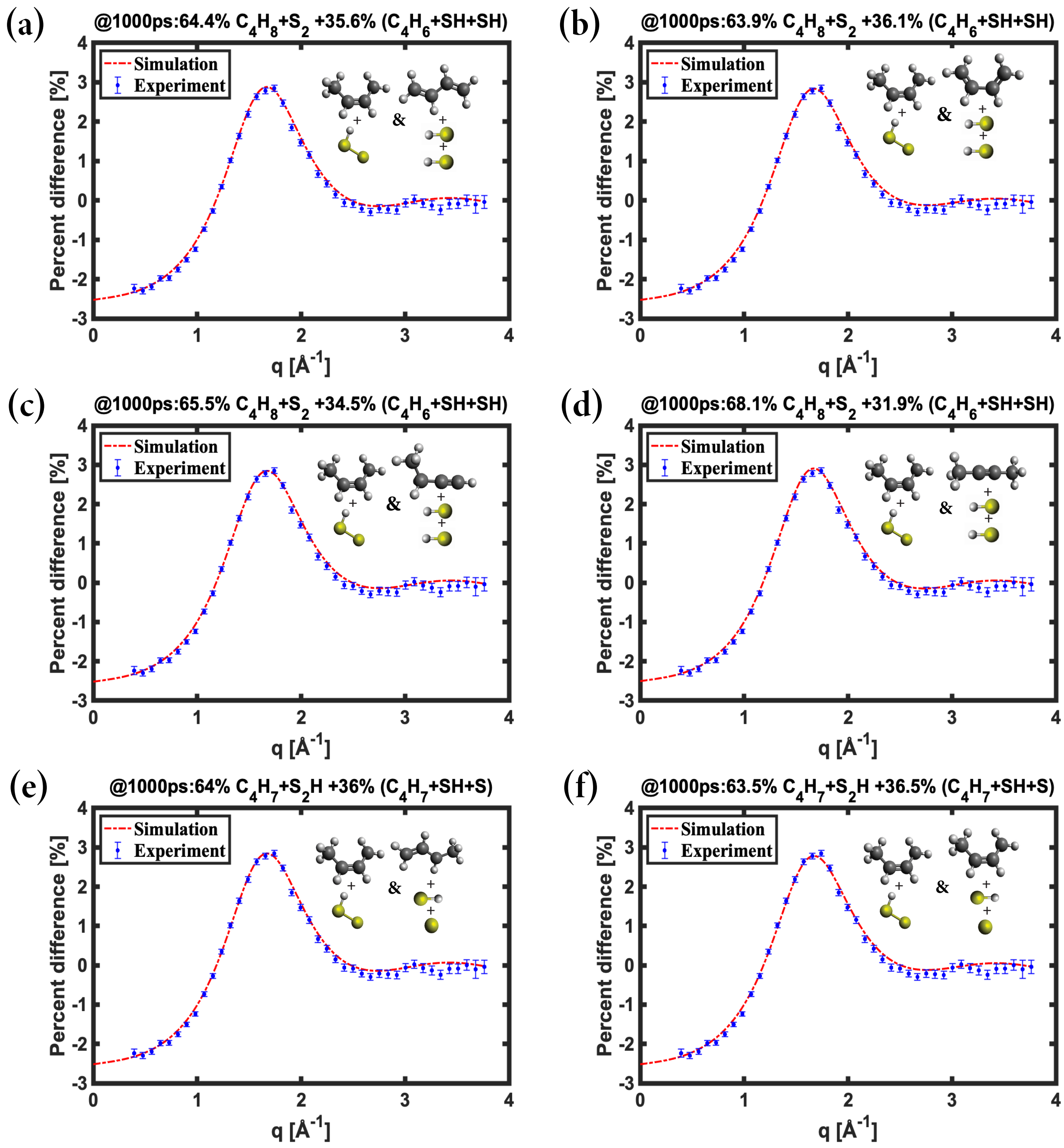

Figure S11 | The fits for the possible structure combinations of C_4H_7 $(geom2) + S_2H$ (FB) and all possible FA-2, (a-f), at 1000 ps. The ratios of FA-2 and FB are all shown on the top of each subplot and obtained through least-square fits between experimental pattern and simulated one with percent excitation $\gamma = 4.51\%$.

Table S2 | Summary of least-square fit results for all possible structural pairs of final photoproducts at 1000 ps.

No.	γ (%)	Ratio @ 1000 ps			
1	4.51	C4H8 (geom1) + S2	65.57	C4H6 (geom1, trans) + SH + SH	34.43
2	4.51		64.71	C4H6 (geom1, cis) + SH + SH	35.29
3	4.51		66.38	C4H6 (geom2) + SH + SH	33.62
4	4.51		69.44	C4H6 (geom3) + SH + SH	30.56
5	4.51		64.84	C4H7 (geom1) + SH + S	35.16
6	4.51		63.93	C4H7 (geom2) + SH + S	36.07
7	4.51	C4H8 (geom2) + S2	66.04	C4H6 (geom1, trans) + SH + SH	33.96
8	4.51		64.95	C4H6 (geom1, cis) + SH + SH	35.05
9	4.51		66.71	C4H6 (geom2) + SH + SH	33.29
10	4.51		70.19	C4H6 (geom3) + SH + SH	29.81
11	4.51		65.13	C4H7 (geom1) + SH + S	34.87
12	4.51		63.94	C4H7 (geom2) + SH + S	36.06
13	4.51	C4H8 (geom3) + S2	66.74	C4H6 (geom1, trans) + SH + SH	33.26
14	4.51		66.03	C4H6 (geom1, cis) + SH + SH	33.97
15	4.51		67.60	C4H6 (geom2) + SH + SH	32.40
16	4.51		70.43	C4H6 (geom3) + SH + SH	29.57
17	4.51		66.04	C4H7 (geom1) + SH + S	33.96
18	4.51		65.30	C4H7 (geom2) + SH + S	34.70
19	4.51	C4H7 (geom1) + S2H	66.40	C4H6 (geom1, trans) + SH + SH	33.60
20	4.51		65.60	C4H6 (geom1, cis) + SH + SH	34.40
21	4.51		67.20	C4H6 (geom2) + SH + SH	32.80
22	4.51		70.19	C4H6 (geom3) + SH + SH	29.81
23	4.51		65.72	C4H7 (geom1) + SH + S	34.28
24	4.51		64.86	C4H7 (geom2) + SH + S	35.14
25	4.51	C4H7 (geom2) + S2H	64.42	C4H6 (geom1, trans) + SH + SH	35.58
26	4.51		63.89	C4H6 (geom1, cis) + SH + SH	36.11
27	4.51		65.47	C4H6 (geom2) + SH + SH	34.53
28	4.51		68.10	C4H6 (geom3) + SH + SH	31.90
29	4.51		63.98	C4H7 (geom1) + SH + S	36.02
30	4.51		63.46	C4H7 (geom2) + SH + S	36.54
Average			66.11		33.89
STEDV			1.94		1.94

It is obvious that all of the given possible structural pairs of photoproducts yield very good agreement with experimental data at the representative end time points, i.e. 700 ps and 1000 ps. The percent excitation of this photochemical reaction is γ =4.51% which is a scalar quantity determined by the kinetics fits of the overall experimental scattering signals. All simulated theoretical scattering patterns shown above are modeled by using the Independent Atom Model

(IAM) and scaled by γ in order to reach the comparable magnitude of experimental signals. The relative ratios between FA-2 and FB are treated as adjustable parameters in each least-square fit.

In summary, taking all listed possible structural pairs at 700 ps and at 1000ps into account, the average percentages of FA-2 and FB are 35% $\pm$ 2%, and 65% $\pm$ 2%, respectively.

3. The structures of the photochemical reaction transients and photoproducts

Table S3 | One possible structure of FA-1 (C_4H_8S) optimized at the B3LYP/6-311+G(d) level of theory shown in Cartesian coordinates

Element	x (Å)	y (Å)	z (Å)
C	-0.00924	-0.04438	0.09783
C	1.32669	0.61915	0.24935
C	2.44416	0.25597	-0.38213
C	3.76513	0.93720	-0.21684
S	4.95079	-0.20530	0.65174
H	-0.76448	0.66288	-0.26394
H	0.03088	-0.88341	-0.60137
H	-0.37245	-0.42255	1.06033
H	1.36658	1.46665	0.93505
H	2.42863	-0.59787	-1.05876
H	4.19938	1.17792	-1.18990
H	3.66427	1.85722	0.36093
H	6.08546	0.43041	0.27660

Table S4 | One possible structure of FA-1 (C_4H_7S) optimized at the B3LYP/6-311+G(d) level of theory shown in Cartesian coordinates

Element	x (Å)	y (Å)	z (Å)
C	0.00825	-0.05654	0.11575
C	1.34393	0.60453	0.26101
C	2.44117	0.28755	-0.43601
C	3.77164	0.95863	-0.25143
S	4.73425	-0.18948	0.80395
H	-0.76284	0.66697	-0.17283
H	0.02607	-0.85154	-0.63364
H	-0.31702	-0.49363	1.06708
H	1.40742	1.41009	0.99328
H	2.41214	-0.53139	-1.15212
H	4.29020	1.07902	-1.20562
H	3.67217	1.93171	0.23108

Table S5 | One possible structure (C_4H_7) of hydrocarbon skeletons both for FA-2 and FB optimized at the B3LYP/6-311+G(d) level of theory shown in Cartesian coordinates

Element	x (Å)	y (Å)	z (Å)
C	-5.62923	-0.14204	0.54081
C	-4.55835	0.67511	0.21861
C	-5.65405	-1.51821	0.41819
C	-4.54981	2.16141	0.35798
H	-6.52794	0.34371	0.92354
H	-3.65218	0.20815	-0.16517
H	-6.52968	-2.09489	0.69067
H	-4.79603	-2.06817	0.04432
H	-3.75044	2.49944	1.03072
H	-5.49779	2.53980	0.74988
H	-4.36398	2.65627	-0.60453

Table S6 | Another possible structure (C_4H_7) of hydrocarbon skeletons both for FA-2 and FB optimized at the B3LYP/6-311+G(d) level of theory shown in Cartesian coordinates

Element	x (Å)	y (Å)	z (Å)
C	-1.74504	3.11057	-0.02552
C	-2.73807	2.08011	-0.10635
C	-2.54191	0.75814	-0.04275
C	-1.23907	0.03256	0.12833
H	-0.70229	2.89727	0.10213
H	-2.03008	4.14218	-0.09471
H	-3.75393	2.41818	-0.23370
H	-3.41242	0.12672	-0.12113
H	-0.38723	0.69698	0.17774
H	-1.07621	-0.65602	-0.69632
H	-1.25048	-0.56227	1.03781

Table S7 | One possible structure (C_4H_8 conformer 1) of hydrocarbon skeletons for FB optimized at the B3LYP/6-311+G(d) level of theory shown in Cartesian coordinates

Element	x (Å)	y (Å)	z (Å)
C	1.30798	2.14122	1.66558
C	0.47137	2.88539	0.94469
C	1.96852	0.87213	1.20401
C	3.50273	0.95782	1.22087
H	1.55884	2.46410	2.67724
H	0.03232	3.79683	1.33857
H	0.19010	2.61037	-0.06916
H	1.65367	0.04617	1.85619
H	1.61924	0.62030	0.19655
H	3.95350	0.01050	0.91055
H	3.86094	1.73991	0.54536
H	3.87789	1.18946	2.22295

Table S8 | One possible structure (C_4H_8 conformer 2) of hydrocarbon skeletons for FB optimized at the B3LYP/6-311+G(d) level of theory shown in Cartesian coordinates

Element	x (Å)	y (Å)	z (Å)
C	1.96722	0.92907	0.88113
C	2.95863	1.65653	1.39609
C	4.42115	1.33587	1.28654
C	0.50470	1.24968	0.99080
H	2.21781	0.02090	0.33017
H	2.70803	2.56466	1.94710
H	4.59218	0.41546	0.72156
H	4.97054	2.14335	0.78833
H	4.87789	1.21309	2.27568
H	0.04764	1.37136	0.00168
H	-0.04443	0.44266	1.49004
H	0.33374	2.17066	1.55488

Table S9 | One possible structure (C_4H_8 conformer 3) of hydrocarbon skeletons for FB optimized at the B3LYP/6-311+G(d) level of theory shown in Cartesian coordinates

Element	x (Å)	y (Å)	z (Å)
C	1.18783	1.43483	0.58646
C	2.47308	1.06989	0.61346
C	3.67077	1.92085	0.92666
C	0.60494	2.79153	0.86231
H	0.45800	0.66556	0.33504
H	2.69898	0.02920	0.38199
H	3.41518	2.95709	1.15412
H	4.22118	1.51870	1.78533
H	4.37380	1.92949	0.08540
H	-0.10754	2.74818	1.69450
H	1.35824	3.54103	1.11083
H	0.04454	3.15906	-0.00538

Table S10 | Possible structures containing sulfur atoms (S_2H and S_2) for FB optimized at the B3LYP/6-311+G(d) level of theory shown in Cartesian coordinates

Element	x (Å)	y (Å)	z (Å)
S	-5.35755	0.52341	0.00000
S	-5.57810	-1.47763	0.00000
H	-6.65140	0.95422	0.00000

Element	x (Å)	y (Å)	z (Å)
S	-0.08746	0.00000	0.00000
S	1.84186	0.00000	0.00000

Table S11 | Possible structures containing sulfur atoms (SH) for FA-1 and FA-2 optimized at the B3LYP/6-311+G(d) level of theory shown in Cartesian coordinates

Element	x (Å)	y (Å)	z (Å)
S	-5.35979	0.51512	0.00000
H	-6.64652	0.94504	0.00000
